# IGLV3-21*01 is an inherited risk factor for CLL through the acquisition of a single-point mutation enabling autonomous BCR signaling

**DOI:** 10.1073/pnas.1913810117

**Published:** 2020-02-11

**Authors:** Palash C. Maity, Mayas Bilal, Marvyn T. Koning, Marc Young, Cornelis A. M. van Bergen, Valerio Renna, Antonella Nicolò, Moumita Datta, Eva Gentner-Göbel, Rob S. Barendse, Sebastiaan F. Somers, Ruben A. L. de Groen, Joost S. P. Vermaat, Daniela Steinbrecher, Christof Schneider, Eugen Tausch, Tamara Bittolo, Riccardo Bomben, Andrea Nicola Mazzarello, Giovanni del Poeta, Wilma G. M. Kroes, J. Tom van Wezel, Katharina Imkeller, Christian E. Busse, Massimo Degano, Tamam Bakchoul, Axel Ronald Schulz, Henrik Mei, Paolo Ghia, Konstantia Kotta, Kostas Stamatopoulos, Hedda Wardemann, Antonella Zucchetto, Nicholas Chiorazzi, Valter Gattei, Stephan Stilgenbauer, Hendrik Veelken, Hassan Jumaa

**Affiliations:** ^a^Institute of Immunology, Ulm University, 89081 Ulm, Germany;; ^b^Department of Hematology, Leiden University Medical Center, 2333 ZA Leiden, The Netherlands;; ^c^Department of Internal Medicine III, Ulm University Hospital, 89081 Ulm, Germany;; ^d^Clinical and Experimental Onco-Hematology Unit, Centro di Riferimento Oncologico di Aviano, Istituto di Ricovero e Cura a Carattere Scientifico (IRCCS), 33081 Aviano, Italy;; ^e^Karches Center for Oncology Research, The Feinstein Institute for Medical Research, Northwell Health, Manhasset, NY 11030;; ^f^Division of Hematology, S. Eugenio Hospital and University of Tor Vergata, 00144 Rome, Italy;; ^g^Department of Clinical Genetics, Leiden University Medical Center, 2333 ZA Leiden, The Netherlands;; ^h^Department of Pathology, Leiden University Medical Center, 2333 ZA Leiden, The Netherlands;; ^i^B Cell Immunology, German Cancer Research Center, 69120 Heidelberg, Germany;; ^j^Biocrystallography Unit, Division of Immunology, Transplantation and Infectious Diseases, IRCCS San Raffaele Scientific Institute, 20132 Milan, Italy;; ^k^Transfusion Medicine, Medical Faculty of Tübingen and Center for Clinical Transfusion Medicine, Universitätsklinikum Tübingen, 72076 Tübingen, Germany;; ^l^Mass Cytometry Lab, German Rheumatism Research Center (DRFZ), a Leibniz Institute, 10117 Berlin, Germany;; ^m^Division of Experimental Oncology, Università Vita-Salute San Raffaele, 20132 Milan, Italy;; ^n^Institute of Applied Biosciences, Centre for Research and Technology Hellas, 57001 Thessaloniki, Greece;; ^o^Department of Hematology, Oncology, Clinical Immunology, and Rheumatology, Saarland University Medical School, 66421 Homburg/Saar, Germany;; ^p^José Carreras Institute for Immunology and Gene Therapy, Saarland University Medical School, 66421 Homburg/Saar, Germany

**Keywords:** chronic lymphocytic leukemia (CLL), B cell antigen receptor (BCR), autonomous BCR signaling, immunoglobulin allele IGLV3-21*01

## Abstract

The prognosis of chronic lymphocytic leukemia (CLL) depends on different markers, including cytogenetic aberrations, oncogenic mutations, and mutational status of the immunoglobulin (Ig) heavy-chain variable (IGHV) gene. The number of IGHV mutations distinguishes mutated (M) CLL with a markedly superior prognosis from unmutated (UM) CLL cases. In addition, B cell antigen receptor (BCR) stereotypes as defined by IGHV usage and complementarity-determining regions (CDRs) classify ∼30% of CLL cases into prognostically important subsets. Subset 2 expresses a BCR with the combination of IGHV3-21–derived heavy chains (HCs) with IGLV3-21–derived light chains (LCs), and is associated with an unfavorable prognosis. Importantly, the subset 2 LC carries a single-point mutation, termed R110, at the junction between the variable and constant LC regions. By analyzing 4 independent clinical cohorts through BCR sequencing and by immunophenotyping with antibodies specifically recognizing wild-type IGLV3-21 and R110-mutated IGLV3-21 (IGLV3-21^R110^), we show that IGLV3-21^R110^–expressing CLL represents a distinct subset with poor prognosis independent of IGHV mutations. Compared with other alleles, only IGLV3-21*01 facilitates effective homotypic BCR–BCR interaction that results in autonomous, oncogenic BCR signaling after acquiring R110 as a single-point mutation. Presumably, this mutation acts as a standalone driver that transforms IGLV3-21*01–expressing B cells to develop CLL. Thus, we propose to expand the conventional definition of CLL subset 2 to subset 2L by including all IGLV3-21^R110^–expressing CLL cases regardless of IGHV mutational status. Moreover, the generation of monoclonal antibodies recognizing IGLV3-21 or mutated IGLV3-21^R110^ facilitates the recognition of B cells carrying this mutation in CLL patients or healthy donors.

## Significance

CLL is characterized by autonomous B cell receptor (BCR) signaling. CLL subsets are empirically defined by sequence similarities of the BCR heavy chain. However, in the unfavorable subset 2, an acquired mutation (termed R110) in the light chain stimulates autonomous BCR signaling. This study demonstrates that the oncogenic R110 mutation dictates the unfavorable prognosis and is not restricted to the conventional subset 2. Interestingly, carriers of a particular light-chain allele (IGLV3-21*01) are predisposed to develop CLL because this allele enables autonomous BCR signaling by R110 as a single-point mutation. Monoclonal antibodies permit convenient screening for R110-expressing CLL, showing that it is the largest immunologically defined CLL subset and an example of functional rather than empirical CLL subclassification.

The most prevalent form of leukemia among adults in the Western world, namely chronic lymphocytic leukemia (CLL), originates from an indolent type of clonal expansion of B cells (1, 2). About 80% of all CLL cases are diagnosed in patients >60 y old (3). The clinical course of CLL varies widely and is associated with distinct recurrent cytogenetic aberrations, gene mutations, and sequence characteristics of the clonal B cell antigen receptor (BCR) expressed by CLL cells (4–12). Specifically, the sequence homology of the immunoglobulin (Ig) heavy-chain variable (IGHV) segment of the BCR heavy chain (HC) to its most closely related germline IGHV segment has been identified empirically as an important prognostic parameter. A categorical cutoff of 98% sequence homology of the CLL IGHV to its germline variant distinguishes the so-called unmutated (UM) and mutated (M) CLL cases. The UM-CLL cases have a strikingly inferior prognosis compared with M-CLL (4, 5). In addition, an important role for the BCR in CLL pathogenesis and aggressiveness is suggested by the observation that ∼30% of all CLL cases, predominantly UM-CLL, can be grouped into so-called BCR stereotypes (13, 14). These stereotypes, also referred to as CLL subsets, are defined by similarities in the BCR HC sequence, specifically by particular IGHV and Ig heavy chain junctional (IGHJ) genes and the complementarity-determining region (CDR)3 sequence created by variable, diversity, and junctional (VDJ) recombination (13, 15, 16). Moreover, each of these subsets is associated with a distinctive clinical course (13–15, 17). Thus, both M-/UM-CLL and subset classifications serve as important markers for disease prognosis. However, this classification requires extensive sequence characterization after initial clinical identification. For example, CLL subset 2 is defined by a BCR HC composed of the IGHV3-21 and IGHJ6 genes with a relatively short CDR3 of 9 amino acids (13). Even though CLL subset 2 cases are mostly identified as M-CLL, they are associated with a poor prognosis similar to UM-CLL cases (13, 17). Additionally, CLL subset 2 is also known to express a light chain (LC) of the lambda isotype that utilizes the IGLV3-21 gene. Expression of IGLV3-21 has also been associated with poor prognosis of CLL (18), although no mechanistic explanation of this observation has been provided (14).

Our group has identified antigen-independent, autonomous BCR signaling as the mechanistic basis for the pathogenic role of the BCR in virtually all cases of CLL (19). Subsequently, exemplary structural crystallographic analyses for CLL subsets 2 and 4 have revealed homotypic interactions between BCR heterodimers as the mechanistic basis for this BCR activation (20). Most importantly, the indispensable R110 residue for this homotypic BCR–BCR interaction originates through nonsynonymous somatic hypermutation (SHM) of the germline G110 residue in the IGLJ segment, and reversion of R110 into G110 abrogates autonomous BCR signaling (20).

Here we report comprehensive characterization of CLL and healthy B cells expressing the R110-mutated IGLV3-21 LC (termed IGLV3-21^R110^) by extensive BCR sequencing, development of IGLV3-21– and IGLV3-21^R110^–specific antibodies, and mass cytometry analyses. We demonstrate the poor prognosis of IGLV3-21^R110^–expressing CLL and propose to replace the conventionally defined CLL subset 2 with this “subset 2L.” With 8 to 18% of CLL cases among different cohorts, subset 2L is a CLL subset defined by functional immunology and represents the largest CLL subset recognized so far. Finally, we identify the IGLV3-21*01 allele as an inherited risk factor to develop CLL subset 2L.

## Results and Discussion

### Monoclonal Antibodies Reveal a High Frequency of IGLV3-21^R110^.

**Fig. 1.**
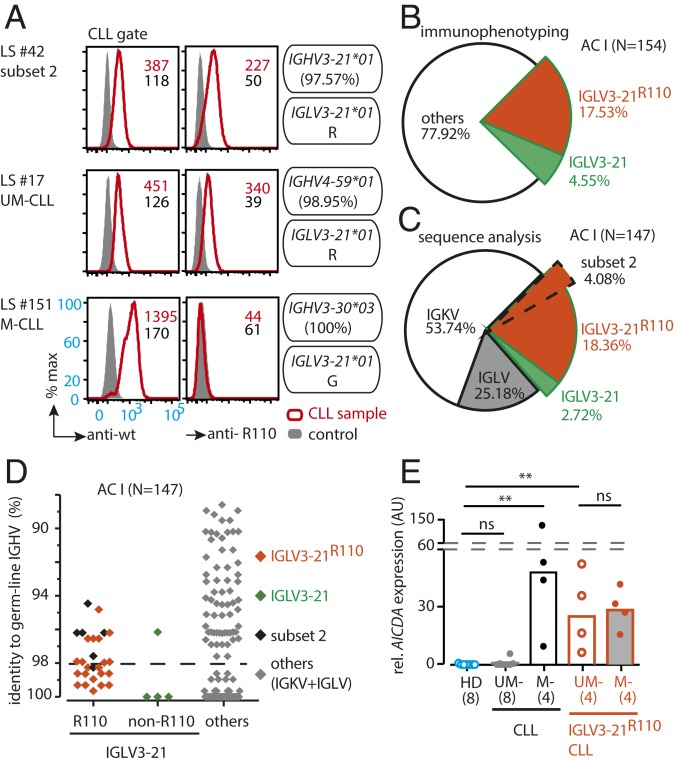
Rapid identification of light-chain IGLV3-21 and IGLV3-21^R110^ from CLL cases. (*A*) Exemplary immunophenotyping histograms to detect wild-type and mutated light chains derived from the IGLV3-21 segment in a CLL subset 2 (LS #42), a UM-CLL (LS #17), and an M-CLL (LS #151) case. Commonly, CLL subset 2 is associated with IGLV3-21–derived LCs carrying R110 as a single-point mutation at the variable–constant region junction. The R110 mutation is referred to as IGLV3-21^R110^. The antibodies recognize wt IGLV3-21 variants and the mutated IGLV3-21^R110^ and are referred to as anti-wt IGLV3-21 and anti–IGLV3-21^R110^, respectively. The expressed IGHV and IGLV alleles and their mutational statuses are indicated alongside. Histograms (red line) show the expression of IGLV3-21 and IGLV3-21^R110^ using fluorescently labeled anti-wt and anti-R110 antibodies, respectively. The plotted cells are pregated for CLL population by CD19 and CD5 expression after excluding dead cells. The control (gray-filled) CLL sample expresses a non–IGLV3-21 LC. Median fluorescence intensities of anti-wt and anti-R110 binding are indicated within the plots. (*B*) Pie chart of the immunophenotyping results depicting the proportion of IGLV3-21– (green) and IGLV3-21^R110^– (red) positive cases in a CLL cohort (*n* = 154). (*C*) Pie chart of IGLV/IGKV sequencing results within the same CLL cohort (*n* = 147), revealing the frequency of IGLV3-21 (green), IGLV3-21^R110^ (red) including CLL subset 2 (dashed area), as well as other IGLVs (gray) and IGKVs (white). (*D*) Scatterplot of the IGHV mutational status of 147 CLL cases having sequence results grouped by different IGLV segments as follows: IGLV3-21^R110^ cases (orange), IGLV3-21 cases (green), and others (gray). The subset 2 CLL cases (black) are depicted within IGLV3-21^R110^–positive cases. The dashed line indicates the conventional 98% cutoff of IGHV sequence homology to its germline variant that distinguishes UM- and M-CLL cases. While <98% IGHV sequence homology defines an M-CLL case, ≥98% IGHV sequence homology defines an aggressive UM-CLL case. (*E*) Bar graph of relative *AICDA* expression (AU) analyzed by qRT-PCR of IGLV3-21^R110^–negative (black) and IGLV3-21^R110^–positive (orange) cases, both of which are subgrouped into UM- (open bars) and M-CLL (gray-filled) cases according to IGHV mutational status, and compared with healthy donor (blue) samples by using the 2-tailed Mann–Whitney *U* test. The plot depicts a median bar along with the individual sample values, and the numbers of samples per group are depicted below. ns, nonsignificant; **P < 0.01.

We generated 2 highly specific monoclonal antibodies to characterize wild-type (wt) IGLV3-21 and mutated IGLV3-21^R110^ LC expression in primary CLL samples (Fig. 1*A* and *SI Appendix*, Fig. S1 *A* and *B*). One recognizes IGLV3-21 variants irrespective of the R110 mutation and is referred to as anti-wt IGLV3-21 (anti-wt) while the other specifically recognizes the mutated IGLV3-21^R110^ and is referred to as anti–IGLV3-21^R110^ (anti-R110). Subsequently, we performed immunophenotyping and determined the frequency of the IGLV3-21^R110^ LC as compared with IGLV3-21 (Fig. 1*A* and *SI Appendix*, Fig. S1*B*) in analysis cohort (AC) I consisting of 154 CLL patients, of which complete informative follow-up data and mutational analyses were available for 122 cases (*SI Appendix*, Tables S1–S6). By analyzing the entire AC I (*N* = 154), we found 34 CLL samples (22.08%) that express IGLV3-21 (Fig. 1*B*). The majority (27 of 34, or 17.53%) of these IGLV3-21 CLL samples possessed an IGLV3-21^R110^ LC, whereas only 7 (4.55%) were negative for the anti-R110 staining (Fig. 1*B*).

In parallel, IGV gene sequencing of 147 cases (of 154 cases from AC I) confirmed the distribution of IGLV3-21 and IGLV3-21^R110^ as determined by immunophenotyping (Fig. 1*C* and *SI Appendix*, Fig. S1*C*). The sequence analysis also revealed that only 6 (4.08%) of the 27 IGLV3-21^R110^–expressing cases belonged to stereotypic CLL subset 2 as defined by IGHV3-21 with a characteristic CDR3 sequence (Fig. 1 *C* and *D*) (13, 18, 20). Several other IGHV genes were found to be effectively pairing with the IGLV3-21 LC (*SI Appendix*, Table S2), indicating that CLL subset 2 represents a minor subgroup of IGLV3-21^R110^–expressing CLL, while the majority have hitherto not been recognized as an immunobiologically related CLL subset. The homology of all IGHV segments of the entire group of IGLV3-21^R110^–expressing cases to their respective germline sequences varied between 94.4 and 99.7% (Fig. 1*D* and *SI Appendix*, Table S2). In contrast, the unmutated IGLV3-21–expressing cases were mostly (3/4) the UM-CLL type based on a cutoff of 98% IGHV homology. Furthermore, the IGLV3-21^R110^–expressing cases predominantly expressed activation-induced cytidine deaminase (*AICDA*), an enzyme initiating SHM (Fig. 1*E*). Since a single IGLV3-21^R110^ mutation could theoretically represent the only mutation required to initiate CLL development, acquisition of additional Ig mutations is nonessential and could hence occur to a variable degree. This scenario explains why IGLV3-21^R110^–expressing CLL straddles the conventionally defined UM- and M-CLL categories, although the origin of IGLV3-21^R110^–expressing CLL biologically requires SHM (20). In contrast, the unmutated IGLV3-21–expressing cases were mostly (3/4) a UM-CLL type based on a cutoff of 98% IGHV homology (Fig. 1*D* and *SI Appendix*, Table S2).

To reduce possible sampling bias, we extended our study and analyzed the additional AC II (*SI Appendix*, Table S7) consisting of 134 CLL patients, all expressing the lambda light chain (IGL). Immunophenotyping and sequencing analyses revealed that 23 and 10 CLL cases expressed mutated IGLV3-21^R110^ (17.16%) and unmutated IGLV3-21 (7.46%), respectively (*SI Appendix*, Fig. S1*D*). As expected, only 4 cases (4/23) were stereotypic CLL subset 2, confirming the fact that CLL subset 2 represents a minor subgroup of IGLV3-21^R110^–expressing CLL. The IGLV3-21^R110^–expressing cases remained uniformly distributed through M- and UM-CLL classification according to IGHV identity (*SI Appendix*, Fig. S1*E* and Table S8).

In contrast, the unmutated IGLV3-21–expressing cases were predominantly (7/10) the UM-CLL type. Similarly, AC III, AC IV, and AC V (*SI Appendix*, Tables S9–S12) confirmed the above findings that IGLV3-21^R110^ CLL is found within M- and UM-CLL and that IGLV3-21^R110^ is not restricted to CLL subset 2, as different IGHV-derived HCs can pair with IGLV3-21^R110^ in CLL pathogenesis. Intriguingly, AC V, originating from the US population, represents very high frequency IGLV3-21^R110^ (14 of 15) as well as CLL subset 2 (13 of 15) cases (*SI Appendix*, Table S12). In contrast, AC IV, originating from the Greek population, shows only 4 CLL subset 2 patients among 14 identified IGLV3-21^R110^ cases (*SI Appendix*, Table S11). Perhaps this difference is attributable to the prevalence of CLL subset 2 on different continents. Together, these data show that IGLV3-21 is overrepresented in CLL and that most cases present as mutated IGLV3-21^R110^ without belonging to CLL subset 2.

### IGLV3-21 and IGLV3-21^R110^ CLLs Are Uniform by Single-Cell Sequence Analysis.

**Fig. 2.**
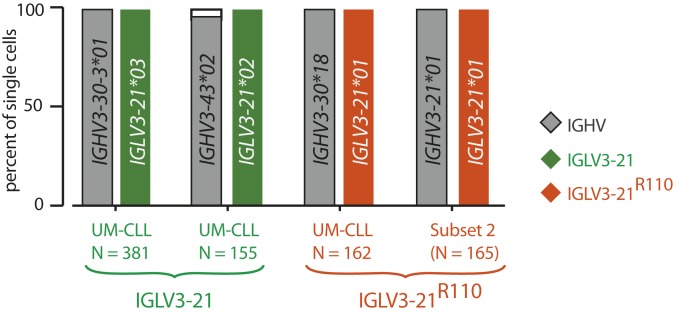
Clonality of IGLV3-21^R110^ CLL cases at the single-cell level. Horizontal bar graph of IGHV and IGLV allele usage determined by single-cell paired HC and LC sequence analyses of 2 exemplary IGLV3-21 (green) and 2 exemplary IGLV3-21^R110^ (orange) CLL cases. Numbers of analyzed single cells are individually depicted. The unique IGHV and IGLV genes and alleles for each case are depicted inside the bars.

Since every IGLV3-21^R110^–expressing case expresses *AICDA* independent of M-CLL or UM-CLL classification (Fig. 1*E*), it is conceivable that these CLL cases are heterogeneous by IGLV3-21 sequence and might possess both unmutated IGLV3-21 and mutated IGLV3-21^R110^ subpopulations. To exclude such subclonal variability, we performed single-cell HC and LC sequencing on exemplary IGLV3-21– and IGLV3-21^R110^–expressing cases (Fig. 2). CLL samples were sorted by fluorescence-activated cell sorting (FACS) (*SI Appendix*, Fig. S2*A*), and the sorted cells were confirmed for IGLV3-21 and IGLV3-21^R110^ expression by anti-wt and anti-R110 staining, respectively (*SI Appendix*, Fig. S2*B*) and plated as single cells. In the subset 2 case, clonal homogeneity was indicated by LC sequences that almost exclusively carried the predicted IGLV3-21^R110^ sequence, and by expression of identical pairs of HC and LC sequences. Similarly, the HC and LC identities of the IGLV3-21–expressing CLL case were found to be uniform at the single-cell level.

Thus, the comprehensive sequencing result of the IGLV3-21– and IGLV3-21^R110^–expressing cells within the respective CLL cases confirms the clonal distribution of the R110 mutation and emphasizes the specificities of the anti-wt and anti-R110 antibodies used for immunophenotyping.

### IGLV3-21^R110^ Defines a Clinically Aggressive CLL Phenotype.

**Fig. 3.**
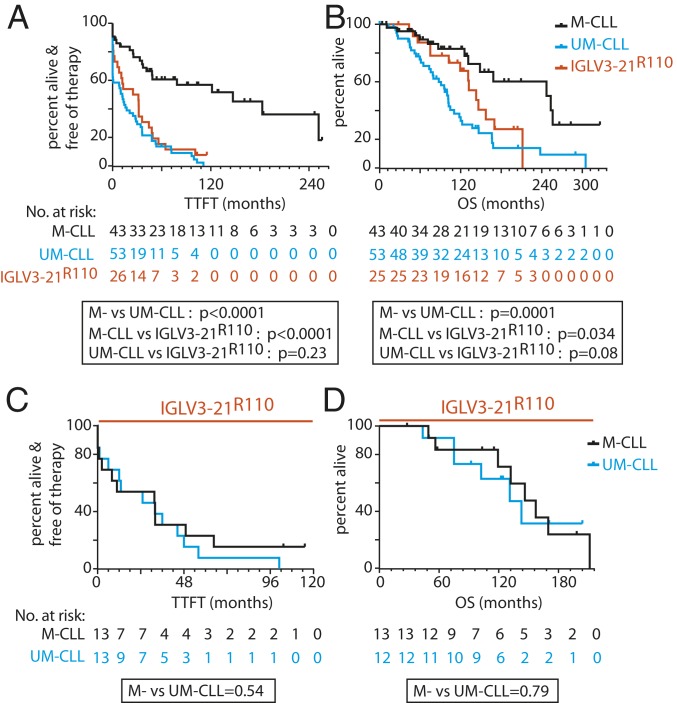
IGLV3-21^R110^ is associated with decreased TTFT and OS (AC I; *n* = 122). (*A*) Kaplan–Meier analysis for treatment-free survival from diagnosis of IGLV3-21^R110^–positive CLL patients compared with IGLV3-21^R110^–negative M-CLL and UM-CLL patients classified by IGHV identity. (*B*) Kaplan–Meier analysis for overall survival of IGLV3-21^R110^–positive CLL patients compared with IGLV3-21^R110^–negative M-CLL and UM-CLL patients. (*C*) Kaplan–Meier analysis for treatment-free survival from diagnosis of IGLV3-21^R110^–positive CLL patients according to IGHV mutational status. (*D*) Kaplan–Meier analysis for OS from diagnosis of IGLV3-21^R110^–positive CLL patients according to IGHV mutational status. All data are from AC I, and the depicted *P* values were obtained from log-rank (Mantel–Cox) analyses.

Next, we investigated whether IGLV3-21^R110^ expression alone identified CLL cases with poor prognosis irrespective of IGHV mutational status or assignment to subset 2. Indeed, for those AC I cases with sufficient outcome information (*n* = 122), the IGLV3-21^R110^–expressing CLL patients required early treatment and had inferior overall survival (OS) than IGLV3-21^R110^–negative M-CLL patients (Fig. 3 *A* and *B*). The required time to first treatment (TTFT) of IGLV3-21^R110^–expressing CLL patients was very similar to IGLV3-21^R110^–negative UM-CLL, and their OS was not significantly different. When IGLV3-21^R110^–expressing CLL patients were separated according to IGHV mutational status, both TTFT and OS (Fig. 3 *C* and *D*) were virtually identical.

Despite the aggressive clinical course, the majority of IGLV3-21^R110^–expressing CLL cases from AC I carried the prognostically favorable del13q14 genetic abnormality (21, 22), whereas the unfavorable del17p or del11q22 genetic abnormality (12, 23) occurred infrequently in CLL cases expressing IGLV3-21^R110^ (*SI Appendix*, Table S2). Multivariable analysis confirmed the adverse impact of IGLV3-21^R110^ on both clinical outcome parameters in comparison with the remaining M-CLL (*SI Appendix*, Tables S3 and S4). In contrast, del17p lost its impact on OS upon multivariable analysis. Moreover, IGHV mutational status had no prognostic relevance in IGLV3-21^R110^–expressing CLL since the prognostic influence of the R110 mutation remained significant when the IGLV3-21^R110^–expressing CLLs were split into IGHV mutated and unmutated cases (*SI Appendix*, Tables S5 and S6).

With respect to genes recurrently mutated in CLL, targeted sequencing of 100 B lymphoma-associated genes revealed *NOTCH1* mutations in IGLV3-21^R110^–expressing CLL at an apparently similar frequency to published cases (Fisher’s exact test: *P* = 0.52) (11). However, *TP53* (*P* = 0.016), *ATM* (*P* = 0.008), and *SF3B1* (*P* < 0.0001) appeared to be more frequently mutated in IGLV3-21^R110^–expressing CLL (*SI Appendix*, Table S2). IGLV3-21^R110^–expressing CLL also carried mutations in genes encoding epigenetic modifiers such as CREBBP, KMT2D, and EP300 (*SI Appendix*, Table S2) that are associated with oncogenesis of germinal center-type lymphomas (24–26).

Next, we analyzed another independent AC II consisting of 134 CLL patients expressing IGL (*SI Appendix*, Table S7) and assessed the prognostic severity of IGLV3-21^R110^ (*n* = 23) cases compared with IGLV3-21 (*n* = 10) cases (*SI Appendix*, Fig. S3*A*). Indeed, the TTFT and OS of the IGLV3-21^R110^ CLL patients were significantly shorter as compared with IGLV3-21 patients, and the remaining IGLV patients (*n* = 102) remained intermediate between IGLV3-21^R110^ and IGLV3-21. The TTFT and OS of these IGLV3-21^R110^ CLL patients (*n* = 66) were identical to those of IGLV3-21–negative UM-CLL (*n* = 36) patients (*SI Appendix*, Fig. S3*B*). Similarly, allocating the IGLV3-21^R110^ CLL patients according to their IGHV mutational status and comparing the outcome revealed that M- and UM-CLL subgroup OSs were virtually identical (*SI Appendix*, Fig. S3*C*). Additionally, 10 (45.4%) of the 22 IGLV3-21^R110^ CLL cases carried the otherwise prognostically favorable del13q14 genetic abnormality (*SI Appendix*, Table S8) as a standalone aberration, confirming our previous finding with AC I (*SI Appendix*, Tables S2–S6). In summary, IGLV3-21^R110^ has an unfavorable impact on CLL prognosis irrespective of CLL-associated genomic aberrations and IGHV mutational status.

**Fig. 4.**
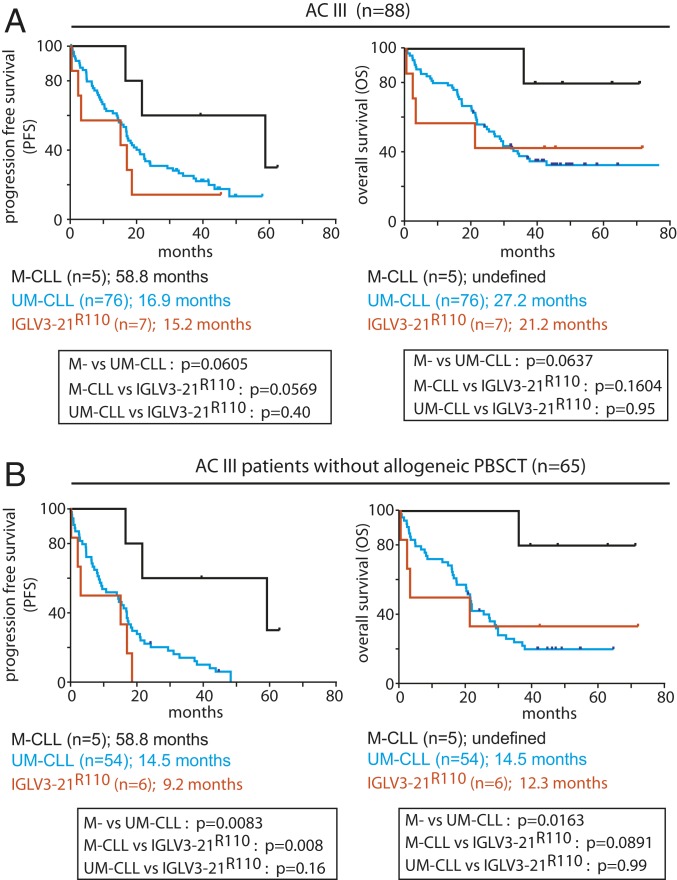
IGLV3-21^R110^ is as severe as UM-CLL cases within a high-risk cohort (AC III; *n* = 90). (*A*) Kaplan–Meier analysis for progression-free survival (*Left*) and overall survival (*Right*) of IGLV3-21^R110^–positive CLL patients compared with IGLV3-21^R110^–negative M-CLL and UM-CLL patients classified by IGHV identity. (*B*) Similar Kaplan–Meier analyses as *A*, but for a patient who received no allogeneic PBSCT. All data are from AC III, and the depicted *P* values were obtained from log-rank (Mantel–Cox) analyses.

As IGLV3-21 seems to be overrepresented in high-risk CLL patients, we analyzed 90 high-risk patients from AC III (CLL2O trial; *SI Appendix*, Table S9). The CLL2O sample cohort was a multicenter, prospective phase II trial to study the efficacy of alemtuzumab (anti-CD52 antibody) and optional allogeneic peripheral blood stem cell transplantation (allo-PBSCT) (10, 27). Presumably due to the inclusion criteria with an emphasis on CLL with del17p and refractory *TP53* mutation, only 7 cases expressed mutated IGLV3-21^R110^ (7.78%) while only 1 case expressed unmutated IGLV3-21 (*SI Appendix*, Fig. S4 and Table S10). Therefore, the total IGLV3-21 frequency in AC III was only 8.89%, but the IGLV3-21^R110^ CLL cases were distributed through M-CLL (3 cases) and UM-CLL (4 cases) classification (*SI Appendix*, Fig. S4) (18, 28). Similarly, different IGHV genes were found to be effectively pairing with the IGLV3-21^R110^ LC and only 2 of the 7 IGLV3-21^R110^ cases were annotated as conventional subset 2, confirming again that subset 2 represents only a minor subgroup of IGLV3-21^R110^–expressing CLL (*SI Appendix*, Table S10). Despite the low patient numbers, IGLV3-21^R110^ CLL patients may have progressed earlier after treatment and had inferior OS than IGLV3-21–negative M-CLL (*n* = 5) within the same cohort (Fig. 4). When restricting the analysis to patients not receiving allo-PBSCT, the outcome of the remaining 6 IGLV3-21^R110^ CLL cases was inferior compared with M-CLL in terms of progression-free survival (PFS), possibly inferior with respect to OS, and similar to UM-CLL (Fig. 4*B*).

Taken together, these results from a prospective multicenter trial confirm that IGLV3-21^R110^ CLL represents a clinically aggressive group even within a select high-risk CLL cohort (AC III), and it is solely defined by LC identity regardless of IGHV family, mutational status, and stereotypy.

### IGLV3-21^R110^ Cellular Phenotype Resembles UM-CLL.

Despite the clinical aggressiveness, IGLV3-21^R110^–expressing CLL cases are found within M-CLL as well as UM-CLL (Fig. 1*D* and *SI Appendix*, Fig. S1*E*). To further investigate the molecular mechanisms, we developed an extensive multiparametric mass cytometry phenotyping pipeline (*SI Appendix*, *Methods and Materials*), also known as CyTOF analyses, and investigated the cellular phenotype of IGLV3-21^R110^ CLL as compared with M- and UM-CLL (*SI Appendix*, Table S13). For each M- and UM-CLL subgroup, we analyzed 5 nonstereotypic CLL samples and analyzed B cells isolated from 5 healthy donors (HDs) as control. Of note, unlike standard cytometry analyses that measure each sample successively, we performed measurement on multiplexed samples barcoded with isotope-labeled anti–β2-microglobulin (B2M) staining (29, 30). Indeed, the barcoding allowed robust comparative analyses between samples (*SI Appendix*, Fig. S5 *A*–*C*). The inclusion of identical HD samples in each run allowed comparison between samples from different batches.

**Fig. 5.**
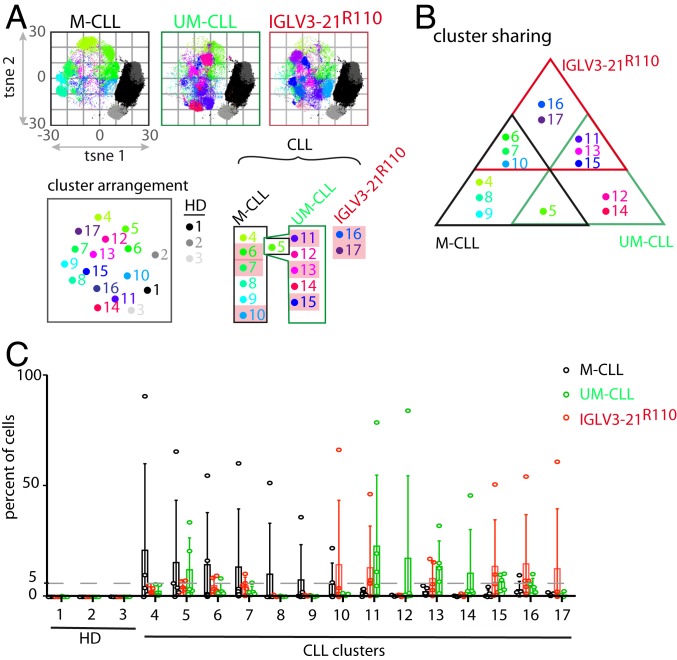
IGLV3-21^R110^ CLL shares cellular phenotypes of both M- and UM-CLL cases. (*A*) PhenoGraph analyses of M-, UM-, and IGLV3-21^R110^–positive CLL cases, all compared with peripheral B cells from HDs. Arrangement, numbering, and coloring of different phenotypic clusters from HD and M- and UM-CLL as well as R110 cases are depicted below the PhenoGraph. For comparison, HD clusters (1 through 3) are depicted in grayscale whereas R110 clusters (6, 7, 10, 11, 13, and 15 through 17) are highlighted purple. (*B*) Distribution and overlap of M- (black), UM- (green), and IGLV3-21^R110^ (red) CLL cases in terms of cluster sharing. (*C*) Interleaved bar graph of the percentage of cells from M-, UM-, and IGLV3-21^R110^ CLL distributed into phenotypic clusters 1 through 17. Data represent the mean ± SD of 5 samples from each of the M-, UM-, and IGLV3-21^R110^ CLL cases. The dashed line represents 5% cutoff.

Upon computing and analyzing the CyTOF results using standard dimensionality reduction algorithms, we identified 17 (1 through 17) unique phenotypic clusters (Fig. 5*A* and *SI Appendix*, Fig. S5*D*). While the healthy B cell isolates were limited to 3 major phenotypically discrete clusters (1 through 3), CLL samples allocated 14 independent phenotypic clusters (4 through 17). Three HD clusters, 1 through 3, resemble major peripheral B cell subpopulations, namely mature naïve (CD23++, CD38+, IgM+, IgD+), immature (CD23+, CD38++, IgM++, IgD+), and memory-like (CD23−, CD38−, IgM++, IgD+) B cells, respectively (*SI Appendix*, Fig. S5*D*).

Expectedly, the M-CLL cells (clusters 4 through 10) were phenotypically different from UM-CLL cells (clusters 11 through 15) and shared only 1 phenotypic cluster (cluster 5; Fig. 5 *A* and *B*). Interestingly, the IGLV3-21^R110^ CLL cells shared phenotypic clusters of both M- and UM-CLL samples (Fig. 5 *B* and *C*). Although sharing 3 clusters from each M- and UM-CLL sample, a closer inspection of individual IGLV3-21^R110^ samples revealed that the major proportion of cells was allocated in UM-CLL clusters (Fig. 5*C* and *SI Appendix*, Fig. S5 *E* and *F*). In addition, both IGHV-mutated (3/5) and IGHV-unmutated (2/5) subgroups of IGLV3-21^R110^ cells were predominantly allocated in UM-CLL phenotypic clusters, suggesting the expected inclination of IGLV3-21^R110^ CLL toward nonstereotypic UM-CLL through shared cellular phenotype. Besides the shared phenotypic clusters, we also identified 2 unique phenotypic clusters (16, 17) of IGLV3-21^R110^ CLL cells. Phenotypically, these 2 clusters possessed elevated CD23 or CD43 combined with reduced CD22 expression, which correlates with proliferating CLL cells (31). Taken together, the phenotypic clustering analyses reveal that the IGLV3-21^R110^ CLL cells are predominantly allocated in UM-CLL classes, as expected from their similar clinical course.

### IGLV3-21^R110^ Stimulates Autonomous Signaling.

**Fig. 6.**
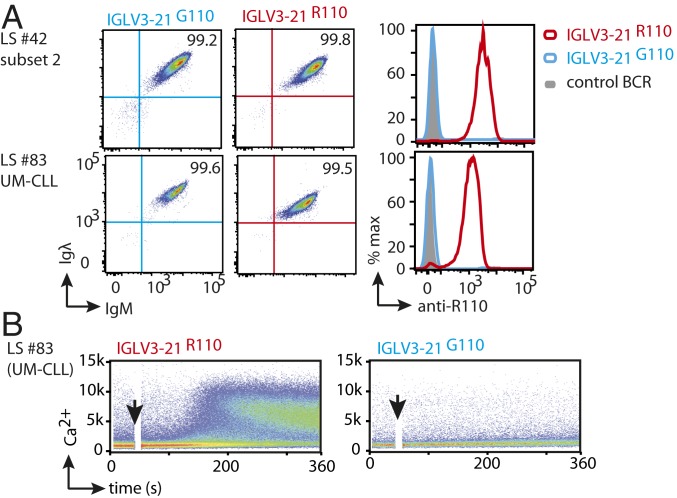
CLL-derived IGLV3-21^R110^ LC boosts autonomous signaling. (*A*, *Left* and *Middle*) BCR expression (IgM HC and Igλ LC) in TKO cells reconstituted with CLL-derived HCs together with reverted IGLV3-21^G110^ (*Left*) or IGLV3-21^R110^ (*Middle*) LC variants. (*A*, *Right*) Overlaid immunophenotyping histograms analyzing the IGLV3-21^R110^ expression in the same reconstituted TKO cell using a fluorescently labeled anti-R110 antibody. (*B*) Exemplary Ca^2+^ release kinetics of the original BCR derived from UM-CLL (LS #83) revealing autonomous BCR signaling and containing an R110-mutated LC (IGLV3-21^R110^; *Left*) as compared with the reverted LC containing a germline G110 (IGLV3-21^G110^; *Right*).

So far, the indispensable role of the IGLV3-21^R110^ mutation for autonomous BCR signaling through homotypic BCR–BCR has only been demonstrated for conventional CLL subset 2 (20). These crystallographic analyses suggested that the unique and short CDR3 in HCs (HCDR3) of subset 2 BCR reinforces correct positioning of LCs for mediating the BCR–BCR interaction (20). However, the variable length and composition of HCDR3 in all CLL cases expressing IGLV3-21^R110^ point to flexibility in the mutual BCR–BCR interaction of R110-positive CLL. Therefore, we examined the role of the R110 mutation in the BCRs derived from non-subset 2 CLL. To analyze autonomous signaling, we expressed BCRs derived from a non-subset 2 (sample ID: LS #83) and a subset 2 CLL (sample ID: LS #42) using retroviral transduction of a previously described cell line derived from RAG2, λ5, and SLP65 triple-knockout (TKO) mice (Fig. 6*A* and *SI Appendix*, Fig. S6*A*) (32, 33). In addition to BCR expression, TKO cells also expressed an ERT2-SLP65 fusion protein for 4-hydroxytamoxifen (4-OHT)–inducible activation of SLP65 function which allowed robust intracellular Ca^2+^ release as a readout for the BCR signaling cascade (19). While the autonomously active BCRs show rapid ligand-independent intracellular Ca^2+^ upon 4-OHT treatments, nonautonomous BCRs require additional ligands such as a cognate antigen or cross-linking antibodies (34). Using this assay, we show that the BCRs derived from both non-subset 2 (LS #83) and subset 2 CLL (LS #42) showed autonomous signaling capacity (Fig. 6*B* and *SI Appendix*, Fig. S6*B*). Expectedly, reverting IGLV3-21^R110^ into the IGLV3-21 LC resulted in defective autonomous signaling (*SI Appendix*, Fig. S6*B*). These data suggest that R110 mutation boosts autonomous BCR signaling and leads to the expansion of the respective B cells in CLL patients.

### The Allele IGLV3-21*01 Is a Risk Factor for IGLV3-21^R110^ CLL.

**Fig. 7.**
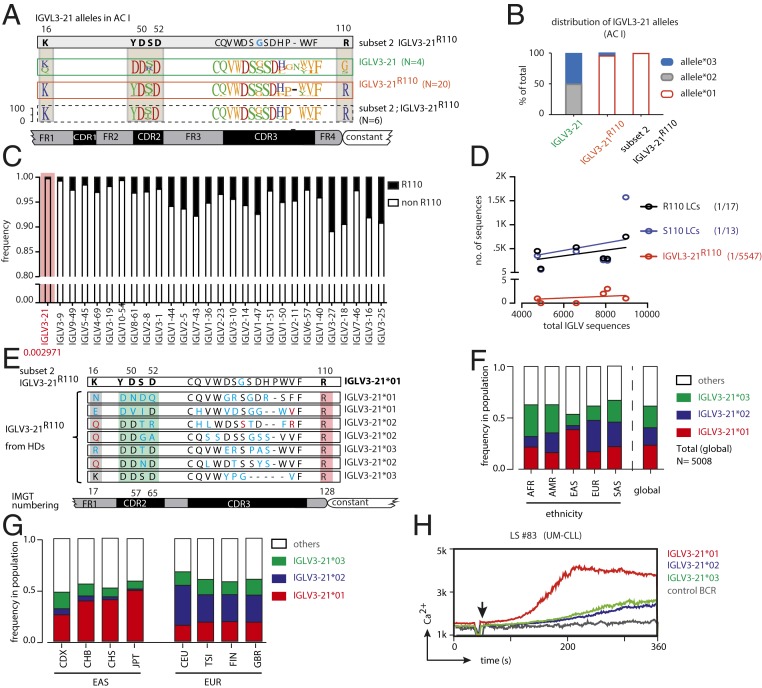
R110-mutated LC allele IGLV3-21*01 is rare in HDs and is causative of autonomous signaling. (*A*) Alignment of LC consensus sequences derived from different groups (IGLV3-21 and IGLV3-21^R110^ CLL cases and CLL subset 2 cases; all from AC I) compared with the reference CLL subset 2 LC, revealing that the K16 residue and the YDSD motif required for homotypic interaction are conserved in virtually all IGLV3-21^R110^ LCs. (*B*) Stacked bar graph for the frequencies of the three different *IGLV3-21* alleles in IGLV3-21– and IGLV3-21^R110^–expressing CLL cases compared with stereotypic CLL subset 2 within AC I patients, revealing the prevalence of the IGLV3-21*01 allele among IGLV3-21^R110^–expressing CLL samples. (*C*) Cumulative stacked frequencies of R110 (black bars) and non-R110 (open bars), which include S110 (*SI Appendix*, Fig. S7*D*) and germline unmutated G110, of different IGLV genes obtained from 6 HDs, demonstrating that IGLV3-21^R110^ has the lowest occurrence (highlighted column), as indicated. Different IGLV genes are sorted by descending frequency of germline residue G110. (*D*) Linear regression of the numbers of R110- and S110-positive LCs and IGLV3-21^R110^ LCs against total IGLV sequences in each of 6 analyzed HDs, revealing that, compared with R110- and S110-positive LCs, IGLV3-21^R110^ LCs have the lowest average expectation independent of sampling size. The obtained average expectations of different LCs are provided along with the labels. (*E*) Sequence alignment of 7 identified IGLV3-21^R110^ rearrangements from the analyzed HDs as compared with a CLL subset 2-derived IGLV3-21^R110^ LC. This alignment reveals that IGLV3-21^R110^ rearrangements originating from HDs lack the combination of the K16 residue (gray-shaded) and YDSD motif (green-shaded) required for homotypic interaction. Residues are numbered according to subset 2-derived IGLV3-21^R110^ (*Upper*) as well as IMGT guidelines (*Lower*). Residues differing from subset 2-derived IGLV3-21^R110^ and those with somatic mutations are indicated by red and blue, respectively. (*F*) Stacked bar graph representing the frequencies of different alleles of the *IGLV3-21* gene among African, American, East Asian, European, and South Asian populations as annotated by the 1000 Genomes Project from the Ensembl GRCh37 genome browser. (*G*) Stacked bar graph of *IGLV3-21* allele frequencies among the subpopulations of the EAS and EUR groups. CDX, Chinese Dai in Xishuangbanna; CEU, Utah residents with northern and western European ancestry; CHB, Han Chinese in Beijing; CHS, southern Han Chinese; FIN, Finnish in Finland; GBR, British in England and Scotland; TSI, Toscani in Italy. (*H*) Comparative analyses of Ca^2+^ release kinetics for a BCR from a UM-CLL (LS #83) carrying the original LC allele IGLV3-21*01 (red) compared with engineered alleles IGLV3-21*02 (blue) and allele IGLV3-21*03 (green) expressing R110. The overlay of median Ca^2+^ release kinetics includes a non-CLL BCR (gray) as control.

The crucial residues required for homotypic BCR–BCR interaction in CLL subset 2 include residues R110 and K16 in one BCR and D50 and D52 of the YDSD motif in a neighboring BCR (20). Notably, the IGLV3-21 gene has 3 major alleles in humans. Of these 3 ImMunoGeneTics (IMGT) annotated alleles of the IGLV3-21 locus, only allele IGLV3-21*01 possesses the prerequisite K16 and YDSD motifs (*SI Appendix*, Fig. S7*A*). Indeed, within AC I, 26 of the 27 IGLV3-21^R110^–positive CLL cases harbor the allele IGLV3-21*01, suggesting that allele IGLV3-21*01 is mechanistically required for the development of IGLV3-21^R110^ CLL (Fig. 7 *A* and *B*). Similarly, all subset 2 cases (*n* = 4) and non-subset 2 IGLV3-21^R110^ CLL cases (*n* = 18) from AC II harbor the allele IGLV3-21*01 (*SI Appendix*, Fig. S7 *B* and *C*). Sequence alignment of all IGLV3-21^R110^ LCs from both AC I and AC II confirmed the prerequisite K16 residue and YDSD motif as well as the association with allele IGLV3-21*01 (Fig. 7*A* and *SI Appendix*, Fig. S7*B*). In contrast, IGLV3-21–expressing CLL cases lacking the R110 mutation harbor alleles IGLV3-21*02 or IGLV3-21*03 (Fig. 7*B* and *SI Appendix*, Fig. S7*C*).

Next, we tested if IGLV3-21^R110^ is exclusive to CLL B cells or whether it exists in B cells from HDs. Based on an unbiased sequence analysis of 41,191 rearranged IGL transcripts from 6 HDs, representing 24,288 unique LC sequences, we found that R110 is a relatively common variant of all IGLVs except IGLV3-21. IGLV3-21^R110^ is strikingly underrepresented in HDs, with the lowest frequency of 1/5,547 compared with all other IGLV segments (1/17 and 1/13) and mutations at the same position (Fig. 7 *C* and *D* and *SI Appendix*, Fig. S7*D*). Interestingly, 6 of 7 identified IGLV3-21^R110^ rearrangements lacked the K16 residue essential for homotypic BCR interaction (Fig. 7*E*). The K16 residue was either lost by nonsynonymous SHM (3/7) or was lacking through usage of allele IGLV3-21*02 encoding a Q16 residue. Moreover, allele IGLV3-21*03 harbors a DDSD motif that may alter the relative positioning of the interacting D50 residue. Furthermore, we analyzed the frequency of the IGLV3-21^R110^–expressing B cell repertoire by single-cell *IGV* sequencing. Expectedly, only 6.38% of the sorted IGL-positive cells from 4 independent HDs expressed IGLV3-21^R110^ and all of them represented either IGLV3-21*02 or IGLV3-21*03 (*SI Appendix*, Fig. S7*E*). Together, these data suggest that the allelic variant IGLV3-21*01 has an intrinsic potential for the generation of BCRs with homotypic interactions and that B cells expressing the IGLV3-21^R110^ variant might be counterselected in HDs.

In addition, we analyzed the occurrence of these 3 common alleles of the *IGLV3-21* gene in healthy human populations worldwide by accessing 1000 Genomes Project data in the Ensembl GRCh37 browser and available tools. Remarkably, the highest frequency of the IGLV3-21*01 allele was recorded among East Asians (EASs) as compared with Africans (AFRs), Americans (AMRs), Europeans (EURs), and South Asians (SASs) (Fig. 7*F*). Moreover, detailed analyses of pertaining subpopulations revealed that the frequencies of the IGLV3-21*01 allele among EUR communities are similar (Fig. 7*G*). In contrast, the frequencies of the IGLV3-21*01 allele among EAS subpopulations differ among different communities and the Japanese population sampled from Tokyo (JPT) showed the highest prevalence (Fig. 7*G*). In agreement with the proposed association of the allele IGLV3-21*01 with CLL, the most frequently expressed light chain in Japanese CLL patients is IGLV3-21 (35).

To examine whether the allele IGLV3-21*01 is required for autonomous signaling, we engineered IGLV3-21^R110^ LC variants using IGLV3-21*02 and IGLV3-21*03. Indeed, BCR containing the IGLV3-21^R110^ LC matching allele IGLV3-21*02 or IGLV3-21*03 showed strikingly reduced autonomous signaling as compared with the original IGLV3-21*01 allele (Fig. 7*H* and *SI Appendix*, Fig. S7*F*). In summary, our data demonstrate that the allele IGLV3-21*01 represents a carrier, which enables efficient BCR–BCR interaction and autonomous signaling upon receiving the transforming R110 point mutation.

Taken together, our study shows that IGLV3-21^R110^ is frequently found in CLL because the allele IGLV3-21*01 of this gene is particularly prone toward acquiring autonomous signaling. Unique positioning of the K16 residue and the YDSD motif predestine the allele IGLV3-21*01 for mediating homotypic BCR–BCR interaction upon acquiring the R110 residue. In contrast, the allelic variants IGLV3-21*02 and IGLV3-21*03 are underprivileged to accomplish homotypic BCR–BCR interaction due to the lack of the prerequisite K16 and the possession of a DDSD motif instead of the YDSD, respectively. To acquire BCR–BCR interaction, the IGLV3-21*02 and IGLV3-21*03 alleles require several additional mutations besides the crucial R110 (20). Consistently, the majority of the IGLV3-21^R110^ CLL cases express the allele IGLV3-21*01. CLL cases expressing either the allele IGLV3-21*02 or IGLV3-21*03 might utilize alternate mechanisms for autonomous BCR signaling independent of R110-mediated interaction. Interestingly, the R110 mutation in HDs associates with IGLV3-21*02 or IGLV3-21*03 but not the IGLV3-21*01 allele, suggesting that the signal-proficient, R110-mutated, IGLV3-21*01 allele might be counterselected.

In summary, by combining structural analyses with IGLV3-21 gene sequences and signaling studies, we identify an Ig allele that increases the risk for CLL development. We describe a scenario of how CLL can develop through a single oncogenic driver mutation acquired in a physiological process, namely AICDA-mediated SHM of the IGLV3-21 gene locus.

Our data show that the unfavorable CLL subtype 2, hitherto empirically defined by sequence characteristics of the BCR HC, should be redefined as subtype 2L based on functional immunopathology and expanded to include all CLL expressing IGLV3-21^R110^, regardless of mutational IGHV status. Subtype 2L comprises around 20% of all CLL cases, thus representing the largest immunologically defined CLL subtype, and carries inferior prognosis despite a high prevalence of the usually favorable del13q14.

Our monoclonal antibodies recognizing the acquired IGLV3-21^R110^ mutation will facilitate convenient recognition of subtype 2L without the necessity for sequence analysis and identification of individuals with increased risk for developing a prognostically unfavorable type of CLL. Furthermore, these antibodies have the potential to develop truly CLL-specific preemptive or clinically indicated therapy that entirely spares nonmalignant B cells.

## Materials and Methods

### Study Populations.

Analysis cohort I: Cryopreserved CLL samples (*n* = 154) were obtained from the Biobank of the Department of Hematology of the Leiden University Medical Center (LUMC) and analyzed (*SI Appendix*, Tables S1–S6). Notably, complete informative follow-up data and mutational analyses were available for 122 patients out of 154 cases in AC I.

AC II: Collection of CLL cases (*n* = 134) expressing λLC was obtained from the Clinical and Experimental Onco-Hematology Unit, Centro di Riferimento Oncologico, IRCCS and analyzed (*SI Appendix*, Tables S7 and S8).

AC III: CLL samples from the CLL2O trial, a multicenter phase II study of alemtuzumab (anti-CD52 antibody) combined with dexamethasone followed by allogeneic stem cell transplantation or alemtuzumab for maintenance (10, 27), were analyzed (*SI Appendix*, Tables S9 and S10). The study was approved by institutional review boards, performed in accordance with the Declaration of Helsinki, and registered at ClinicalTrials.gov (Identifier NCT01392079).

AC IV: CLL samples (*n* = 22) expressing IGLV3-21 were obtained from the Institute of Applied Biosciences at the Centre for Research and Technology Hellas and analyzed (*SI Appendix*, Table S11).

AC V: CLL samples (*n* = 15) expressing IGLV3-21 were obtained from the Karches Center for Oncology Research, The Feinstein Institute for Medical Research, Northwell Health and analyzed (*SI Appendix*, Table S12).

CyTOF analysis panel: CLL samples for mass cytometry (CyTOF) analyses were obtained from the Department of Internal Medicine III, University Hospital Ulm and analyzed (*SI Appendix*, Table S13).

All samples were obtained with informed consent and used in full compliance with institutional regulations. Peripheral blood mononuclear cells from healthy donors were obtained from the Institute for Clinical Transfusion Medicine and Immunogenetics at Ulm University Medical Center and the Center for Clinical Transfusion Medicine, University Medical Center Tübingen. For deep IGLV sequence analyses, HD samples were obtained from the Biobank, LUMC. Detailed protocols are described in *SI Appendix*.

### Antibodies and Immunophenotyping.

Detailed immunophenotyping protocol, reagents, and antibodies are described in *SI Appendix*. Briefly, 100 µL of thawed samples was washed, stained with antibody dilutions, and analyzed by flow cytometry (BD Fortessa).

The monoclonal anti–IGVL3-21 antibodies were generated by ProteoGenix using recombinant IgG containing a CLL subset 2-specific light chain. Both the anti–wild-type IGLV3-21 and the anti–IGLV3-21^R110^ antibodies are IgG2a and Igκ.

### IGV Sequencing and Annotation.

Expressed IGV gene rearrangements from CLL samples and HDs were sequenced by ARTISAN (36) and analyzed by ImMunoGeneTics HighV-QUEST (37). Details are in *SI Appendix*.

### Single-Cell Sorting and IGV Sequencing.

CLL- and HD-derived B cells were sorted as single cells into 384-well plates using a BD FACSAria III. After complementary (c)DNA generation, IGHV, Ig LC Kappa variable, and IGLV transcripts were amplified by Matrix scPCR using the specific primers and barcode extensions as reported previously (38), sequenced on an Illumina MiSeq (2 × 300 bp), and analyzed by sciReptor, version v1.1-5-gaa3ec1b (39).

### Mass Cytometry.

Detailed mass cytometry staining protocol, reagents, and antibodies including their origins, data acquisition, and analysis are described in *SI Appendix*. Briefly, 2 × 10^6^ cells were labeled with Pd and Pt isotope-conjugated B2M antibodies for barcoding prior to pooling (29, 30). Thereafter, the pooled samples were sequentially processed for surface staining, fixation, and permeabilization followed by intracellular staining. Finally, the stained cells were resuspended in Milli-Q water supplemented with EQ 4-element calibration beads, filtered through a 35-μm mesh, and analyzed by the Helios CyTOF instrument. Data were debarcoded, filtered, and gated in FlowJo and analyzed by the open-source R-based integrated mass cytometry analysis platform cytofkit (Bioconductor).

### Calcium Flux Measurement.

Detailed protocols for cloning and expression of BCRs followed by calcium flux analysis were performed as described previously (34, 40). Briefly, the IGHV and IGLV sequences obtained from the CLL sample analyses were cloned into retroviral expression vectors for human µHC and λLC flanked by an internal ribosomal entry sequence followed by split-green fluorescent protein (GFP) reporters. The resulting µHC and λLC plasmids were transfected in retroviral packaging Phoenix cell lines and the culture medium containing the secreted virus was collected after 2 d of transfection. Thereafter, TKO cells expressing ERT2-SLP65 were transduced with virus supernatant by spin-infection method and GFP-expressing BCR-positive cells were analyzed after 2 to 5 d of transduction. Briefly, 2 × 10^6^ transduced cells preloaded with the calcium-sensitive dye Indo-1 (Invitrogen) were analyzed by flow cytometry (BD Fortessa) upon application of 2 µM 4-OHT as described (32).

### RT-PCR.

Total RNA was isolated from healthy donors’ and UM- and M-CLL patients’ peripheral blood mononuclear cells. cDNA was synthesized using the High-Capacity RNA-to-cDNA Kit (Applied Biosystems). Expression of the *AICDA* gene was measured using a TaqMan probe (Assay ID Hs00757808_m1; Thermo Scientific) according to the manufacturer’s protocol.

### Statistical Analysis.

Data plotting and statistical analyses were performed in Prism 7 (GraphPad) and the R software platform. Time from diagnosis to first treatment, progression-free survival, and overall survival were obtained from the patients’ clinical records and compared by the Kaplan–Meier method and log-rank test. The hazard ratios of age and Rai/Binet stage at diagnosis, genetic aberrations, and BCR characteristics were calculated by Cox proportional-hazard regression analyses. All tests were 2-sided, and statistical significance was defined as *P* value < 0.05.

### Data Sharing and Detailed Protocol.

In compliance with institutional regulations, all materials and experimental outcomes will be shared either by public deposit or emails to the corresponding author. Detailed protocols associated with different experiments are amended in *SI Appendix*, *Methods and Materials*. Sequencing results of VDJ and annotations reported in this paper are provided in *SI Appendix*, Tables S1–S13.

## Supplementary Material

Supplementary File

## References

[1] Chen Q., Economic burden of chronic lymphocytic leukemia in the era of oral targeted therapies in the United States. J. Clin. Oncol. 35, 166–174 (2017). 27870563 10.1200/JCO.2016.68.2856 PMC5559889

[2] Hallek M., Chronic lymphocytic leukemia: 2015 update on diagnosis, risk stratification, and treatment. Am. J. Hematol. 90, 446–460 (2015). 25908509 10.1002/ajh.23979

[3] Brenner H., Gondos A., Pulte D., Trends in long-term survival of patients with chronic lymphocytic leukemia from the 1980s to the early 21st century. Blood 111, 4916–4921 (2008). 18309034 10.1182/blood-2007-12-129379

[4] Damle R. N., Ig V gene mutation status and CD38 expression as novel prognostic indicators in chronic lymphocytic leukemia. Blood 94, 1840–1847 (1999). 10477712

[5] Hamblin T. J., Davis Z., Gardiner A., Oscier D. G., Stevenson F. K., Unmutated Ig V(H) genes are associated with a more aggressive form of chronic lymphocytic leukemia. Blood 94, 1848–1854 (1999). 10477713

[6] Rassenti L. Z., ZAP-70 compared with immunoglobulin heavy-chain gene mutation status as a predictor of disease progression in chronic lymphocytic leukemia. N. Engl. J. Med. 351, 893–901 (2004). 15329427 10.1056/NEJMoa040857

[7] Damle R. N., CD38 expression labels an activated subset within chronic lymphocytic leukemia clones enriched in proliferating B cells. Blood 110, 3352–3359 (2007). 17684154 10.1182/blood-2007-04-083832 PMC2200908

[8] Rossi D., Integrated mutational and cytogenetic analysis identifies new prognostic subgroups in chronic lymphocytic leukemia. Blood 121, 1403–1412 (2013). 23243274 10.1182/blood-2012-09-458265 PMC3578955

[9] Pflug N., Development of a comprehensive prognostic index for patients with chronic lymphocytic leukemia. Blood 124, 49–62 (2014). 24797299 10.1182/blood-2014-02-556399 PMC4260976

[10] Stilgenbauer S., Alemtuzumab combined with dexamethasone, followed by alemtuzumab maintenance or allo-SCT in “ultra high-risk” CLL: Final results from the CLL2O phase II study. Blood 124, 1991 (2014).

[11] Puente X. S., Non-coding recurrent mutations in chronic lymphocytic leukaemia. Nature 526, 519–524 (2015). 26200345 10.1038/nature14666

[12] International CLL-IPI Working Group, An international prognostic index for patients with chronic lymphocytic leukaemia (CLL-IPI): A meta-analysis of individual patient data. Lancet Oncol. 17, 779–790 (2016). 27185642 10.1016/S1470-2045(16)30029-8

[13] Agathangelidis A., Stereotyped B-cell receptors in one-third of chronic lymphocytic leukemia: A molecular classification with implications for targeted therapies. Blood 119, 4467–4475 (2012). 22415752 10.1182/blood-2011-11-393694 PMC3392073

[14] Stamatopoulos K., Agathangelidis A., Rosenquist R., Ghia P., Antigen receptor stereotypy in chronic lymphocytic leukemia. Leukemia 31, 282–291 (2017). 27811850 10.1038/leu.2016.322

[15] Stamatopoulos K., Over 20% of patients with chronic lymphocytic leukemia carry stereotyped receptors: Pathogenetic implications and clinical correlations. Blood 109, 259–270 (2007). 16985177 10.1182/blood-2006-03-012948

[16] Murray F., Stereotyped patterns of somatic hypermutation in subsets of patients with chronic lymphocytic leukemia: Implications for the role of antigen selection in leukemogenesis. Blood 111, 1524–1533 (2008). 17959859 10.1182/blood-2007-07-099564

[17] Baliakas P., Clinical effect of stereotyped B-cell receptor immunoglobulins in chronic lymphocytic leukaemia: A retrospective multicentre study. Lancet Haematol. 1, e74–e84 (2014). 27030157 10.1016/S2352-3026(14)00005-2

[18] Stamatopoulos B., The light chain IgLV3-21 defines a new poor prognostic subgroup in chronic lymphocytic leukemia: Results of a multicenter study. Clin. Cancer Res. 24, 5048–5057 (2018). 29945996 10.1158/1078-0432.CCR-18-0133

[19] Dühren-von Minden M., Chronic lymphocytic leukaemia is driven by antigen-independent cell-autonomous signalling. Nature 489, 309–312 (2012). 22885698 10.1038/nature11309

[20] Minici C., Distinct homotypic B-cell receptor interactions shape the outcome of chronic lymphocytic leukaemia. Nat. Commun. 8, 15746 (2017). 28598442 10.1038/ncomms15746 PMC5472768

[21] Calin G. A., Frequent deletions and down-regulation of micro-RNA genes miR15 and miR16 at 13q14 in chronic lymphocytic leukemia. Proc. Natl. Acad. Sci. U.S.A. 99, 15524–15529 (2002). 12434020 10.1073/pnas.242606799 PMC137750

[22] Ouillette P., The prognostic significance of various 13q14 deletions in chronic lymphocytic leukemia. Clin. Cancer Res. 17, 6778–6790 (2011). 21890456 10.1158/1078-0432.CCR-11-0785 PMC3207001

[23] Kröber A., V(H) mutation status, CD38 expression level, genomic aberrations, and survival in chronic lymphocytic leukemia. Blood 100, 1410–1416 (2002). 12149225

[24] Pasqualucci L., Inactivating mutations of acetyltransferase genes in B-cell lymphoma. Nature 471, 189–195 (2011). 21390126 10.1038/nature09730 PMC3271441

[25] Zhang J., Disruption of KMT2D perturbs germinal center B cell development and promotes lymphomagenesis. Nat. Med. 21, 1190–1198 (2015). 26366712 10.1038/nm.3940 PMC5145002

[26] Avnir Y., Structural determination of the broadly reactive anti-IGHV1-69 anti-idiotypic antibody G6 and its idiotope. Cell Rep. 21, 3243–3255 (2017). 29241550 10.1016/j.celrep.2017.11.056 PMC7185437

[27] Steinbrecher D., Telomere length in poor-risk chronic lymphocytic leukemia: Associations with disease characteristics and outcome. Leuk. Lymphoma 59, 1614–1623 (2018). 29063805 10.1080/10428194.2017.1390236

[28] Stamatopoulos K., Immunoglobulin light chain repertoire in chronic lymphocytic leukemia. Blood 106, 3575–3583 (2005). 16076869 10.1182/blood-2005-04-1511

[29] Mei H. E., Leipold M. D., Schulz A. R., Chester C., Maecker H. T., Barcoding of live human peripheral blood mononuclear cells for multiplexed mass cytometry. J. Immunol. 194, 2022–2031 (2015). 25609839 10.4049/jimmunol.1402661 PMC4323739

[30] Schulz A. R., Mei H. E., Surface barcoding of live PBMC for multiplexed mass cytometry. Methods Mol. Biol. 1989, 93–108 (2019). 31077101 10.1007/978-1-4939-9454-0_7

[31] Patten P. E., IGHV-unmutated and IGHV-mutated chronic lymphocytic leukemia cells produce activation-induced deaminase protein with a full range of biologic functions. Blood 120, 4802–4811 (2012). 23071276 10.1182/blood-2012-08-449744 PMC3520620

[32] Meixlsperger S., Conventional light chains inhibit the autonomous signaling capacity of the B cell receptor. Immunity 26, 323–333 (2007). 17331747 10.1016/j.immuni.2007.01.012

[33] Köhler F., Autoreactive B cell receptors mimic autonomous pre-B cell receptor signaling and induce proliferation of early B cells. Immunity 29, 912–921 (2008). 19084434 10.1016/j.immuni.2008.10.013

[34] Iype J., Differences in self-recognition between secreted antibody and membrane-bound B cell antigen receptor. J. Immunol. 202, 1417–1427 (2019). 30683703 10.4049/jimmunol.1800690 PMC6379807

[35] Nakahashi H., Characterization of immunoglobulin heavy and light chain gene expression in chronic lymphocytic leukemia and related disorders. Cancer Sci. 100, 671–677 (2009). 19220298 10.1111/j.1349-7006.2009.01092.x PMC11159379

[36] Koning M. T., ARTISAN PCR: Rapid identification of full-length immunoglobulin rearrangements without primer binding bias. Br. J. Haematol. 178, 983–986 (2017). 27301611 10.1111/bjh.14180

[37] Alamyar E., Duroux P., Lefranc M. P., Giudicelli V., IMGT(®) tools for the nucleotide analysis of immunoglobulin (IG) and T cell receptor (TR) V-(D)-J repertoires, polymorphisms, and IG mutations: IMGT/V-QUEST and IMGT/HighV-QUEST for NGS. Methods Mol. Biol. 882, 569–604 (2012). 22665256 10.1007/978-1-61779-842-9_32

[38] Murugan R., Imkeller K., Busse C. E., Wardemann H., Direct high-throughput amplification and sequencing of immunoglobulin genes from single human B cells. Eur. J. Immunol. 45, 2698–2700 (2015). 26138551 10.1002/eji.201545526 PMC5008140

[39] Imkeller K., Arndt P. F., Wardemann H., Busse C. E., sciReptor: Analysis of single-cell level immunoglobulin repertoires. BMC Bioinformatics 17, 67 (2016). 26847109 10.1186/s12859-016-0920-1 PMC4743164

[40] Übelhart R., Responsiveness of B cells is regulated by the hinge region of IgD. Nat. Immunol. 16, 534–543 (2015). 25848865 10.1038/ni.3141

