# Supplementary material for: IGLV3-21*01 is an inherited risk factor for CLL through the acquisition of a single-point mutation enabling autonomous BCR signaling
Source: Proc Natl Acad Sci U S A. 2020 Feb 11;117(8):4320–7. doi: 10.1073/pnas.1913810117 (PMC7049113; doi:10.1073/pnas.1913810117)
Supplement: Supplementary File [file pnas.1913810117.sapp.pdf]

Supporting Information

Supplement to:

Immunoglobulin allele *IGLV3-21*01* is an inherited risk factor for CLL through the acquisition of a single point mutation enabling autonomous BCR signaling

Palash C. Maity[1], Mayas Bilal[1], Marvyn T. Koning[2], Marc Young[1], Cornelis A.M. van Bergen[2], Valerio Renna[1], Antonella Nicolò[1], Moumita Datta[1], Eva Gentner-Göbel[1], Rob S. Barendse[2], Sebastiaan F. Somers[2], Ruben A.L. de Groen[2], Joost S.P. Vermaat[2], Daniela Steinbrecher[3], Christof Schneider[3], Eugen Tausch[3], Tamara Bittolo[4], Riccardo Bomben[4], Andrea Nicola Mazzarello[5], Giovanni del Poeta[6], Wilma G.M. Kroes[7], J. Tom van Wezel[8], Katharina Imkeller[9], Christian E. Busse[9], Massimo Degano[10], Tamam Bakchoul[11], Axel Ronald Schulz[12], Henrik Mei[12], Paolo Ghia[13], Konstantia Kotta[14], Kostas Stamatopoulos[14], Hedda Wardemann[9], Antonella Zucchetto[4], Nicholas Chiorazzi[5], Valter Gattei[4], Stephan Stilgenbauer[3,15§], Hendrik Veelken[2§], Hassan Jumaa[1§]*

[1] Institute of Immunology, Ulm University, 89081 Ulm, Germany

[2]Department of Hematology, Leiden University Medical Center, 2333 ZA Leiden, The Netherlands

[3]Department of Internal Medicine III, Ulm University Hospital, 89081 Ulm, Germany

[4]Clinical and Experimental Onco-Hematology Unit, Centro di Riferimento Oncologico di Aviano (CRO) IRCCS, 33081 Aviano, Italy

[5]Karches Center for Oncology Research, The Feinstein Institute for Medical Research, Northwell Health, Manhasset, NY, 11030, USA

[6]Division of Hematology, S. Eugenio Hospital and University of Tor Vergata, 00144 Rome, Italy

[7]Department of Clinical Genetics, Leiden University Medical Center, 2333 ZA Leiden, The Netherlands

[8]Department of Pathology, Leiden University Medical Center, 2333 ZA Leiden, The Netherlands

[9]B Cell Immunology, German Cancer Research Center, Im Neuenheimer Feld 280, 69120 Heidelberg, Germany

[10]Biocrystallography Unit, Division of Immunology, Transplantation and Infectious Diseases, IRCCS San Raffaele Scientific Institute, 20132 Milan, Italy

[11]Transfusion Medicine, Medical Faculty of Tübingen and Center for Clinical Transfusion Medicine, Universitätsklinikum Tübingen, 72076 Tübingen, Germany

[12]Mass Cytometry Lab, German Rheumatism Research Center (DRFZ), A Leibniz Institute, Berlin, Germany.

[13]Division of Experimental Oncology, Università Vita-Salute San Raffaele, 20132 Milan, Italy

[14]Institute of Applied Biosciences, Centre for Research and Technology Hellas, 57001 Thessaloniki, Greece

[15]Department of hematology, oncology, clinical immunology and rheumatology, and José Carreras Center for Immuno and Gene therapy, Saarland University Medical School, 66421 Homburg/Saar, Germany

Running Title: High-risk R110 mutated allele *IGLV3-21*01* in CLL.

www.pnas.org/cgi/doi/10.1073/pnas.1913810117

Table of content | Page No.

1. SI Methods and Materials	2-7
2. SI References	8
3. SI Figures	
Fig. S1 - Fig. S7	9-15
4. SI Tables	
Tables S1 – Table S13	16-29

1. SI Methods and Materials

Study population

The study was conducted on the collection of cryopreserved CLL samples from different centers as follows:

Analysis Cohort I (AC I): Cryopreserved CLL samples were obtained from the Biobank of the Department of Hematology of Leiden University Medical Center (LUMC). Among 154 samples, complete informative follow-up as well as different experimental and molecular characterizations were available for 122 CLL patients (outlined in Table S1-S6). Recurrent CLL-associated genetic aberrations were analyzed by fluorescence *in situ* hybridization on interphase nuclei and targeted ion current sequencing.

AC II: Cryopreserved samples, those were immunophenotypically confirmed as IGL expressing cases were obtained from the Clinical and Experimental Onco-Hematology Unit, Centro di Riferimento Oncologico, I.R.C.C.S., Italy. Disease characteristics of 134 IGL expressing CLL patients as well as different experimental and molecular characterizations are outlined in Table S7-S8.

AC III: Cryopreserved samples from the CLL2O trial were used as high risk CLL cohort (Table S9-10). The clinical trial is registered at ClinicalTrials.gov, Identifier: NCT01392079(1, 2).

AC IV: Cryopreserved IGLV3-21-expressing CLL samples, those were immunophenotypically confirmed and assessed by sequencing, were obtained from the Institute of Applied Biosciences the Centre for Research and Technology Hellas, Thessaloniki, Greece (Table S11).

AC V: Cryopreserved IGLV3-21-expressing CLL samples (N=15) were obtained from the Karches Center for Oncology Research, The Feinstein Institute for Medical Research, Northwell Health, Manhasset, NY, USA and were analyzed (Table S12).

CyTOF Analysis panel: For mass-cytometry (CyTOF) analyses, CLL samples were obtained from the Department of Internal Medicine III, University Hospital Ulm. List of CLL samples used in mass-cytometry (CyTOF) analyses and sample related characteristics are outlined in Table S13.

Fresh peripheral blood mononuclear cells (PBMCs) from young healthy blood donors (HDs) were obtained from the Institute for Clinical Transfusion Medicine and Immunogenetics at Ulm University Medical Center and Center for Clinical Transfusion Medicine, University clinic Tübingen, in accordance with respective institutional ethical permissions. For deep IGLV sequence analyses, six samples of healthy stem cell donors were obtained from the Biobank of the Department of Hematology, LUMC. Until further use, PBMCs samples were cryopreserved after Ficoll based enrichment.

For Immunophenotyping analyses, samples were gently thawed as following: approximately 100µL of CLL PBMCs containing 3x10^6 or HDs PBMCs containing 5x10^6 were incubated with 5mL of RPMI 1640 (ThermoFisher) supplemented with 10% FCS (Sigma-Aldrich), 10 units/ml penicillin/streptomycin (ThermoFisher) and 2mM of L-glutamine (ThermoFisher). Thereafter cells were washed with FACS buffer containing 3% FCS in PBS (ThermoFisher). Thereafter cells were incubated in ice for 30 min in 50µL of FACS buffer containing following antibodies: Brilliant Violet 510(BV510)-conjugated anti-

CD19, phycoerythrin-Cy7 (PE-Cy7)-conjugated anti-CD5, Alexa flour 488 (AF488)-conjugated anti-wt (IGLV3-21), AF647-conjugated anti-R110 (IGLV3-21^{R110}) and unconjugated Fc blocker as listed below. For determining the frequency of IGLV3-21^{R110}-expressing B cells in PBMC from HDs, we used the same protocol and following combination of antibodies: BV510 anti-CD19, PE-Cy7 anti-CD5, PE anti-λ and AF647 anti-R110. Afterwards, samples were washed twice with FACS buffer followed by incubation with 1:10,000 diluted Sytox Blue (ThermoFisher) dead cells staining dye in PBS, and immediately analyzed in BD Fortessa LSRII. The results were exported as .fcs file format and analyzed in FlowJo 10.4 (Tree Star Inc.) platform.

List of antibodies

Conjugated antibodies	Applied dilutions	Clone and Isotype	Company and Cat. No.
BV510 anti-CD19	1:100	HIB19; mouse IgG1, κ	Biolegend; 302242
PE-Cy7 anti-CD5	1:100	L17F12; mouse IgG2a, κ	Biolegend; 364008
PE anti-Igλ	1:100	MHL38; mouse IgG2a, κ	Biolegend; 316608
Human BD Fc Block	1:100		BD Bioscience; 564219
AF488 anti-wt (IGLV3-21)	0.06μg/mL	42-1-D1-19; mouse IgG2a, κ	Produced and conjugated using AF488 (cat. No. A20100) and AF647 (cat. No. A37573) reagent from Invitrogen
AF647 anti-R110	0.06μg/mL	16-B2-18; mouse IgG2a, κ	

ARTISAN Sequencing and annotation

Expressed IGV gene rearrangements from the CLL samples as well as HDs were sequenced by template-switching anchored PCR with nested priming (ARTISAN) protocol.(3) Briefly, full-length VDJ and VJ transcripts corresponding to IGHV and IGLV/IGKV sequences were amplified from isolated mRNA (Dynabeads mRNA DIRECT Kit, Invitrogen) by ARTISAN PCR using nested priming on μ, γ, κ, and λ constant regions. ARTISAN amplicons from CLL samples were analyzed by bidirectional Sanger sequencing. BCRs from healthy donors were sequenced on the RSII platform (Pacific Biosciences). The most closely related immunoglobulin V, D, and J segments, somatic mutations, and CDR3 sequences were identified by the ImMunoGeneTics (IMGT) HighV-QUEST tool.(4)

Cell culture

For analyzing the functionality and autonomous signaling, CLL derived BCRs were expressed in TKO cells system as described (5-7). Briefly, TKO cells were derived from a *RAG2*, *λ5* and *SLP65* triple knockout (TKO) mice and Phoenix ECO cells were obtained from ATCC CRL-3214. Both were cultured in Iscove's medium (Biochrom) supplemented with 10 mM L-glutamine (Gibco), 10% heat-inactivated fetal bovine serum (FBS; PAN-Biotech), 100 U /ml penicillin/streptomycin (Gibco) and 50 µM β-mercaptoethanol (Gibco). For culturing TKO cells, the medium was supplemented with supernatant of J558L mouse plasmacytoma cells stably transfected with a murine IL-7 expression vector.

Mass-Cytometry

Antibody conjugates not available from Fluidigm were prepared in-house using the Maxpar antibody labeling kit (Fluidigm) according to the manufacturer's protocol. Briefly, 100µg antibody were mildly reduced with TCEP, lanthanide ions were loaded onto chelate-carrying polymers, and pre-loaded polymers were conjugated to the reduced antibody, washed, and kept in storage buffer. In order to determine the working dilutions, labeled antibodies were tested and titrated for CyTOF experiments according to manufacturer's instructions. Palladium and platinum-labeled antibodies were produced as described before (8-10).

Cryopreserved PBMCs were thawed in a water bath at 37°C, and thereafter diluted with 10 ml of RPMI 1640 medium containing 10% FBS, penicillin-streptomycin and glutamine (all from Sigma). Afterwards, cells were incubated with 1:1000 diluted Cell-ID™ Cisplatin-194Pt (Fluidigm) in 2 mL serum free medium, followed by quenching reaction with serum containing medium, to discriminate between live and dead cells. Cisplatin treated cells were incubated in 10 ml complete medium supplemented with Pierce™ universal nuclease (ThermoFisher) for 10 min at 37°C. At this stage, 2×10^6 cells from each sample were transferred into separate well of a 96 deep-well plate and resuspended in PBS (Rockland Inc.) containing, 0.5% BSA (Sigma), 0.02% NaN3 (Sigma) and 5mM of EDTA, hereafter called cell staining medium (CSM). Cells were then treated with Human BD Fc Block (BD Bioscience) as well as β2-microglobulin (B2M) based combinatorial barcoding reagents as described before (10). Briefly, samples were incubated with two different isotope-labeled anti-B2M (clone: 2M2) antibodies by using a ${}^{5}C_{2}$ scheme from the following isotopes: Pd 104, Pd 106, Pd 108, Pd 110 and Pt 198.

Staining: Barcoded samples were combined together and were incubated in CSM containing required dilutions of isotope labeled antibodies against surface markers for 30 min in ice. Cells were then washed and fixed with 1ml of freshly prepared 1.5% PFA (EMS) in CSM. Fixed cells were washed and cells subjected to methanol (ThermoFisher) permeabilization for 15 min at 4°C, and then washed twice with CSM to remove remaining methanol. For intracellular staining, permeabilized cells were incubated in CSM containing required dilutions of isotope labeled antibodies for 1 hour at RT under continuous

agitation. Thereafter, intracellular-stained cells were incubated overnight in CSM containing 4% PFA and 1:2000 diluted Cell-ID™ Intercalator-Ir (Fluidigm) at 4 °C in order to firmly fix and to discriminate the single cells, respectively.

Data acquisition: after staining and fixation, cells were washed once with CSM and twice with MilliQ™ water. Prior to data acquisition, cells were resuspended at 5×10^6 cells/ml in MilliQ™ water supplemented with EQ™ Four element calibration beads containing cerium (140/142Ce), europium (151/153Eu), holmium (165Ho), and lutetium (175/176Lu), and filtered through a 35µm mesh. Thereafter, cells were acquired using a Helios CyTOF® platform at a rate of 300 events per second.

Data processing, analyses and plotting: The raw measurements were normalized and calibrated using the EQ™ Four element calibration beads and transformed within the Helios system software. The processed FCS3.0 data file generated by Helios system was filtered to remove debris, dead cells and doublets by using paired Cell-ID™ Intercalator-Ir interaclator channels (Ir191 and Ir193) and Cisplatin-194Pt channel vs event length, respectively. Thereafter processed data were debarcoded in FlowJo10.04 based on combinatorial anti-B2M markers to generate individual files for each sample. The cell populations of interest for CLL and healthy donor samples were gated by $CD19^+CD5^+CD3^-$ and $CD19^+CD5^-CD3^-$, respectively.

All filtered and gated data files were then transferred to the cytofkit (Bioconductor) package, an open source R-based integrated mass-cytometry analysis platform, and processed by down-sampling to 5000 cells per sample by ceil method. Data were transformed by cytofAsinh method for all surface markers except CD19, CD5 and CD3. For visualization, results were clustered by Rphenograph method using distance parameter k=30(11), and with maximum number of 1000 iterations, and then plotted as paired t-distributed stochastic neighbor embedding (tsne).

List of metal isotope conjugated antibodies

**Target**	**Clone No.**	**Manufacturer**	**Metal Isotope**	**Isotope conjugation**	**Working Dilution**
CD19	HIB19	Biolegend	Gd 155	In house	1:100
CD5	L17F12	Biolegend	Ho 165	In house	1:100
CD3	OKT3	Biolegend	In 115	In house	1:50
CD20	2H7	Biolegend	Eu 153	In house	1:20
CD43	84-3CI	Fluidigm	Nd 150	purchased	1:20
CD23	EBVCS-5	Biolegend	Gd 158	In house	1:20
CD22	HIB22	Biolegend	Yb 172	In house	1:20
CD45	HI30	Fluidigm	Y 89	purchased	1:100
CD38	HIT2	Biolegend	Sm 152	In house	1:20
CD40	5C3	Fluidigm	Nd 142	purchased	1:20

CD29	TS2/16	Biolegend	Nd 144	In house	1:50
CD49d	9F10	Biolegend	Nd 143	In house	1:20
CXCR4	12G5	Fluidigm	Lu 175	purchased	1:100
CXCR5	J252D4	Biolegend	Yb 176	In house	1:50
IgM	G20-127	BD Bioscience	Yb 174	In house	1:50
IgD	IA6-2	Biolegend	Gd 156	In house	1:100
pSyk (Y319/352)	17a	Fluidigm	Yb 171	purchased	1:20
pPLC γ2 (Y759)	REA341	Miltenyi	Dy 164	In house	1:20
pAKT (S473)	REA359	Miltenyi	Er 167	In house	1:20
pMEK1 (S298)	REA375	Miltenyi	Dy 163	In house	1:20
pNF-κB (S529)	REA 348	Miltenyi	Gd 160	In house	1:50
pSTAT1 (Y701)	REA 159	Miltenyi	Dy 161	In house	1:20
pSTAT3 (S727)	REA 324	Miltenyi	Tm 169	In house	1:50

Plasmids and Retroviral transduction

For inducible activation of SLP65, TKO cells were retrovirally transduced with ERT2-SLP65 construct carrying reporter tdTomato as described (5, 7). *IGHV* and *IGLV* chains were cloned and expressed as human IgM using the bi-molecular fluorescent complementation (BiFC) vector system as described (5, 7). Retroviral transduction was performed as described previously(6, 7). Briefly, Phoenix ECO cells were transfected using GeneJuice Transfection Reagent (Merck Millipore) as recommended by the manufacturer's protocol. Supernatants were collected 48 h post-transfection and used for transduction of TKO cells. Spin-infection of TKO cells was carried out at 1800 rpm and 37°C for 3 h using 10 μg/ml Polybrene Transfection Reagent (Merck Millipore).

Calcium Flux Measurement

For analyzing the functionality and autonomous signaling capacity of the expressed BCRs, cells were analyzed for calcium flux as described previously (5, 6). Briefly, 4-hydroxy tamoxifen (4-OHT)-inducible ERT2-SLP65 expressing TKO cells were reconstituted with HC and LC vectors carrying complementing fragments of a split GFP reporter. Approximately $2x10^6$ cells were preloaded with the calcium-sensitive dye Indo-1 (Invitrogen) for 45 minutes at 37°C. Thereafter, cells were washed and analyzed by flow cytometry (BD Fortessa) for autonomous calcium flux upon application of 2μM 4-OHT.

SI References

1. Steinbrecher D, *et al.* (2018) Telomere length in poor-risk chronic lymphocytic leukemia: associations with disease characteristics and outcome. *Leuk Lymphoma* 59(7):1614-1623.
2. Stilgenbauer S, *et al.* (2014) Alemtuzumab Combined with Dexamethasone, Followed By Alemtuzumab Maintenance or Allo-SCT in "ultra High-risk" CLL: Final Results from the CLL2O Phase II Study. *Blood* 124(21):1991-1991.
3. Koning MT, *et al.* (2017) ARTISAN PCR: rapid identification of full-length immunoglobulin rearrangements without primer binding bias. *Br J Haematol* 178(6):983-986.
4. Alamyar E, Duroux P, Lefranc MP, & Giudicelli V (2012) IMGT((R)) tools for the nucleotide analysis of immunoglobulin (IG) and T cell receptor (TR) V-(D)-J repertoires, polymorphisms, and IG mutations: IMGT/V-QUEST and IMGT/HighV-QUEST for NGS. *Methods Mol Biol* 882:569-604.
5. Kohler F, *et al.* (2008) Autoreactive B cell receptors mimic autonomous pre-B cell receptor signaling and induce proliferation of early B cells. *Immunity* 29(6):912-921.
6. Meixlsperger S, *et al.* (2007) Conventional light chains inhibit the autonomous signaling capacity of the B cell receptor. *Immunity* 26(3):323-333.
7. Ubelhart R, *et al.* (2015) Responsiveness of B cells is regulated by the hinge region of IgD. *Nat Immunol* 16(5):534-543.
8. Mei HE, Leipold MD, & Maecker HT (2016) Platinum-conjugated antibodies for application in mass cytometry. *Cytometry A* 89(3):292-300.
9. Mei HE, Leipold MD, Schulz AR, Chester C, & Maecker HT (2015) Barcoding of live human peripheral blood mononuclear cells for multiplexed mass cytometry. *J Immunol* 194(4):2022-2031.
10. Schulz AR & Mei HE (2019) Surface Barcoding of Live PBMC for Multiplexed Mass Cytometry. *Methods Mol Biol* 1989:93-108.
11. Levine JH, *et al.* (2015) Data-Driven Phenotypic Dissection of AML Reveals Progenitor-like Cells that Correlate with Prognosis. *Cell* 162(1):184-197.

Fig. S1

Immunophenotyping of IGLV3-21 and IGLV3-21^{R110} using anti-wt and anti-R110 antibodies

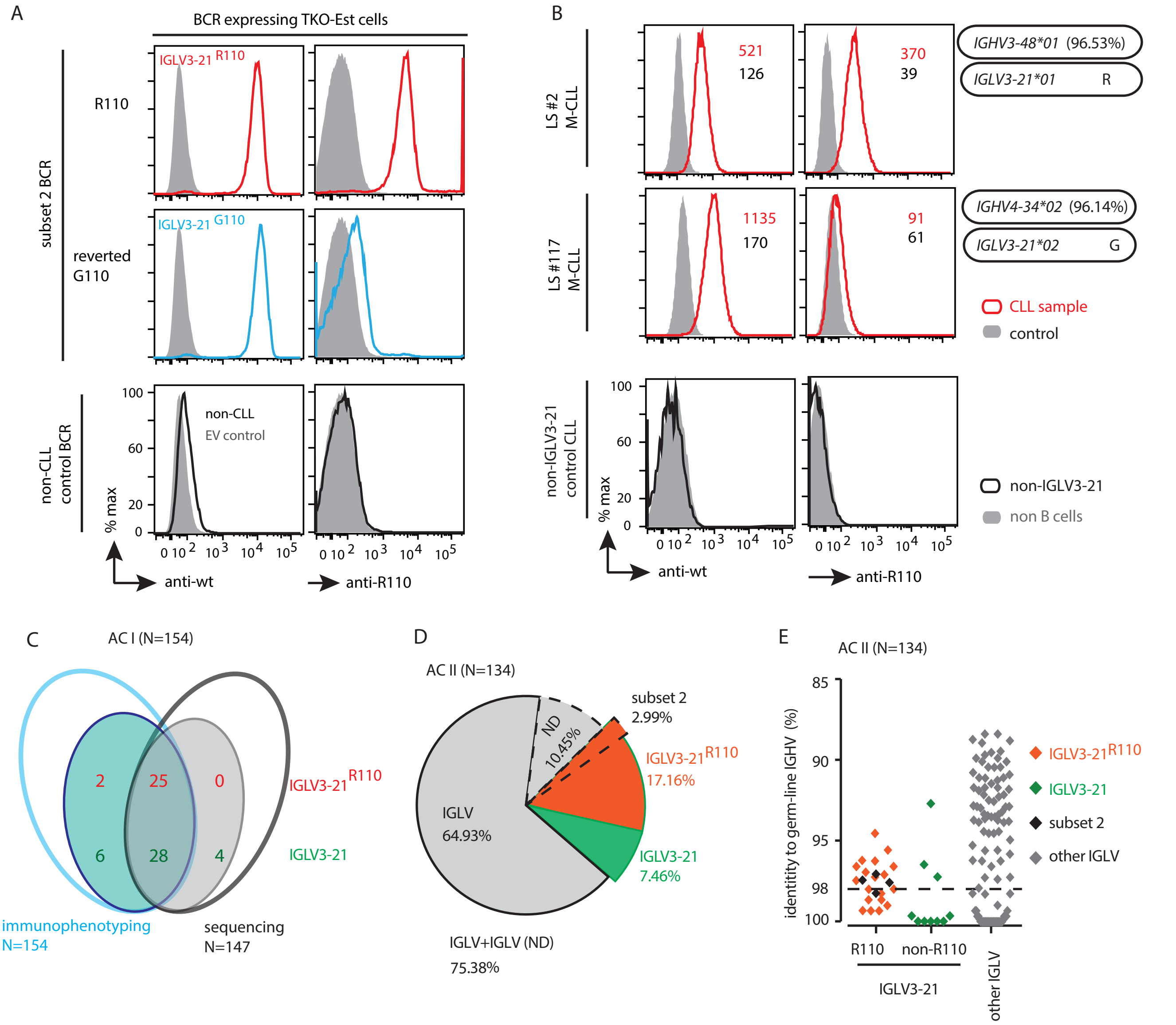

Panel A demonstrates the specificities of the anti-wt and anti-R110 antibodies detecting the expression of mutated IGLV3-21^{R110} (red) and reverted IGLV3-21^{G110} (blue) LCs, respectively. Histograms show immunophenotyping of BCR reconstituted TKO cells expressing CLL subset 2 derived HCs together with either mutated IGLV3-21^{R110} (red, top panels) or reverted IGLV3-21^{G110} (blue, middle panels) LCs compared to TKO cells expressing non-CLL BCRs (black, bottom panels). Each histogram includes an EV transfection control (gray solid).

Panel B, top and middle panels represent exemplary immunophenotyping histograms of two M-CLL cases (LS #2 and LS #117) and for each CLL case the expressed IGHV allele and IGLV allele are provided along with their mutation status. Bottom panels show the staining of control (black) CLL sample expressing a non-IGLV3-21 LC. Histograms (red line) show the expression of IGLV3-21 and IGLV3-21^{R110} using fluorescently labeled anti-wt and anti-R110 antibodies, respectively. Median fluorescence intensities (MFIs) of anti-wt and anti-R110 binding are indicated within the plots. The plotted cells are pre-gated for CLL population by CD19 and CD5 expressions after excluding the dead cells. The control (gray filled) CLL sample expresses a non-IGLV3-21 LC.

Panel C represents Venn diagram showing overlap between the immunophenotyping (N=154) and sequencing (N=147) analyses for identifying IGLV3-21 and IGLV3-21^{R110} –positive cases in AC I as shown in Fig. 2B and Fig. 2C.

Panel D shows Pie chart of IGLV sequencing results from AC II (N=134), revealing the frequency of IGLV3-21 (green part), IGLV3-21^{R110} (red part) including CLL subset#2 (dashed part) as well as other IGLV (gray part). ND refers to not determined.

Fig. S2

Identification of IGLV3-21^{R110} at single cell level

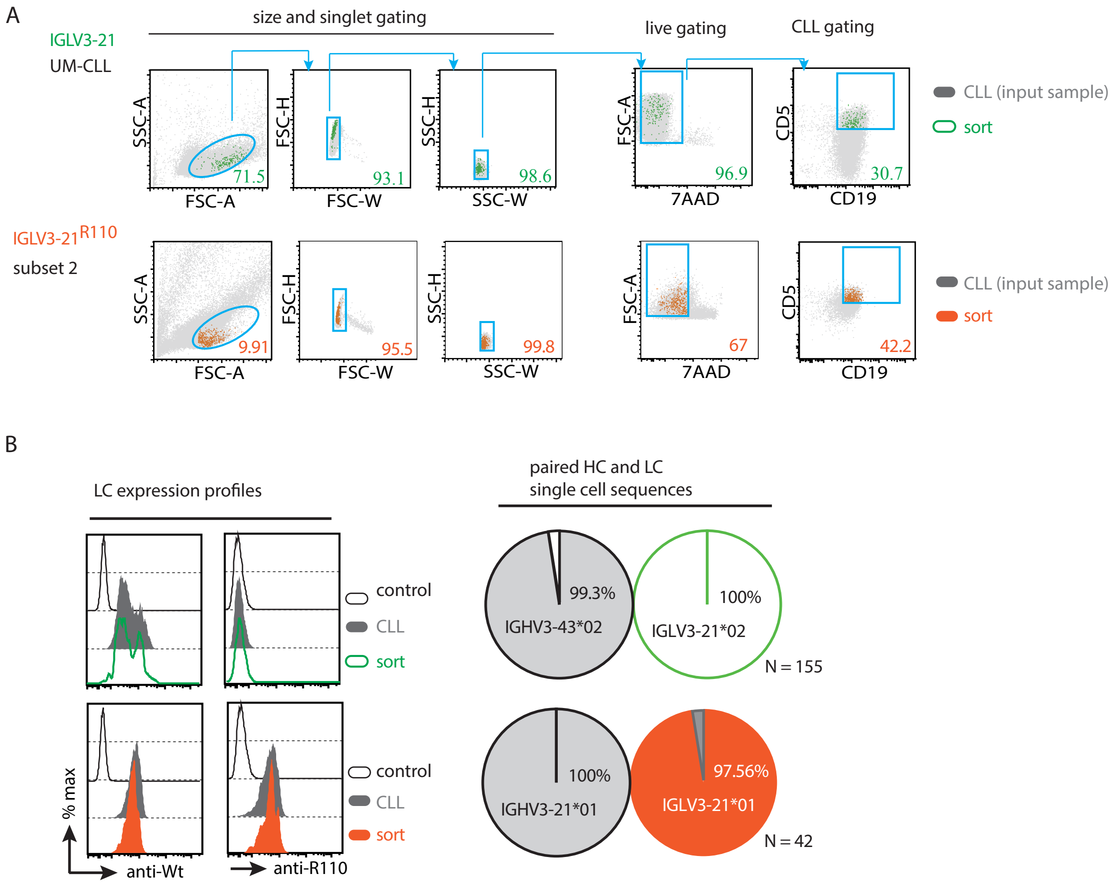

Panel A, left to right, shows the gating strategy and selection of single cells from CLL samples for sorting prior to IGHV and IGLV sequencing.

Panel B, left, histograms show exemplary analyses of IGLV3-21 (top) and IGLV3-21^{R110} (bottom) CLL cases by immunophenotyping with anti-wt and anti-R110 antibodies during cell sorting for single cell IGHV and IGLV sequencing. Each plot represents expression in all sorted single cells (green or orange lines) and the actual CLL sample (black line) compared to non-related IGLV3-21 negative CLL as control (gray filled). The plotted cells are pre-gated for CLL population by CD19 and CD5 expressions after excluding the dead cells. Right, Pie chart of paired IGHV and IGLV sequencing results from sorted single cells.

Fig. S3

IGLV3-21^{R110} is severe compared to other IGLV (AC II; N=134)

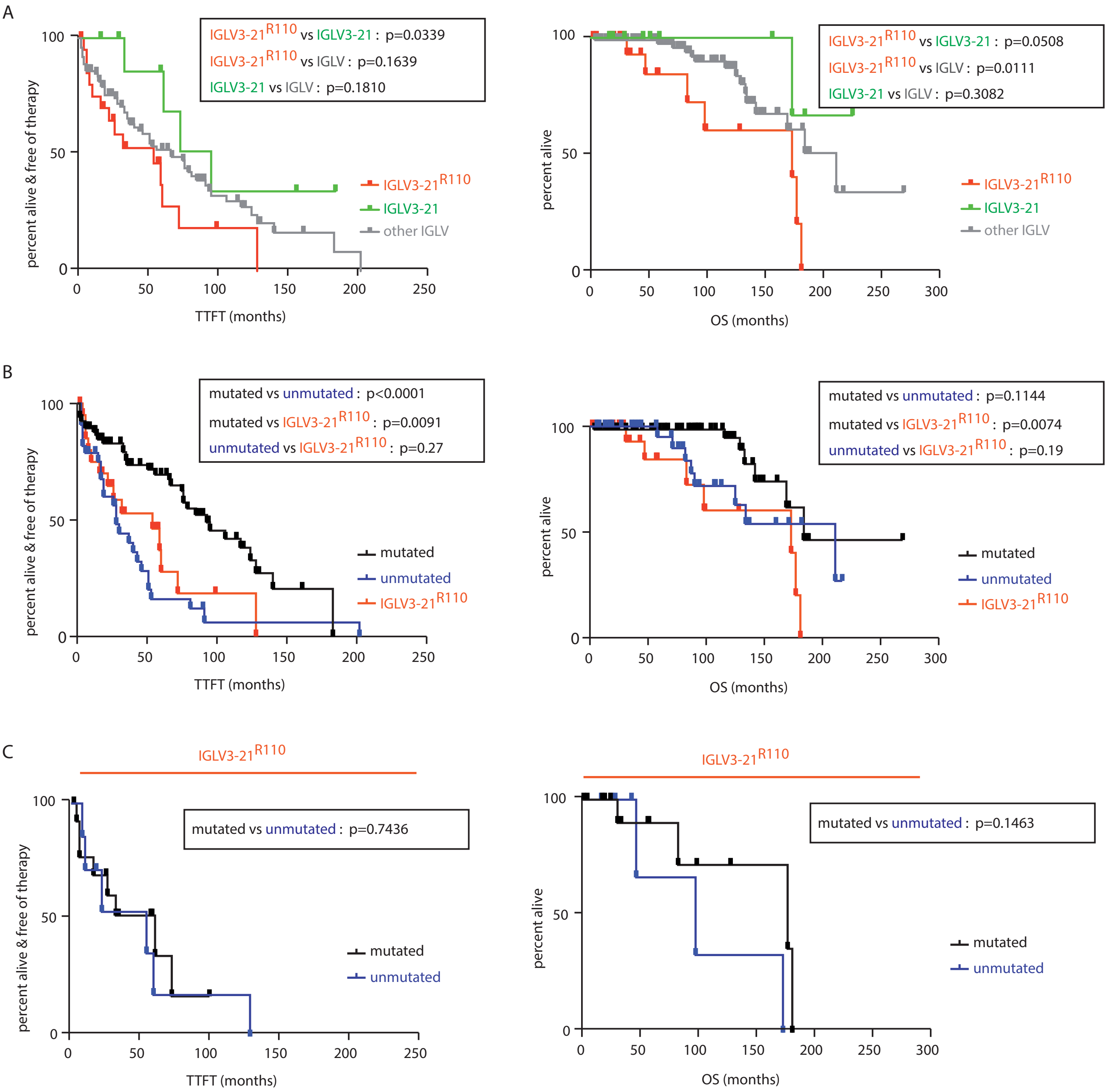

Left to right, Panel A shows Kaplan–Meier analyses for treatment-free survival (TTFT) and overall survival (OS) of 23 IGLV3-21^{R110} (orange) and 10 IGLV3-21 (green) CLL patients compared to other 101 IGLV (gray) cases in AC II (N=134).

Panel B shows same Kaplan–Meier analyses for TTFT and OS of 23 IGLV3-21^{R110}–positive CLL patients in AC II, but compared to IGLV3-21-negative 66 M-CLL and 35 UM-CLL patients classified by IGHV identity.

Panel C shows similar Kaplan–Meier analyses for TTFT and OS, but compared between 15 M-CLL and 8 UM-CLL cases of IGLV3-21^{R110}–positive CLL. All data in Panel A-C are from AC II, and the depicted p values were obtained from Log-rank (Mantel-Cox) analyses.

Fig. S4

Distribution of IGLV3-21^{R110} in high-risk CLL cases (ACIII; N=90)

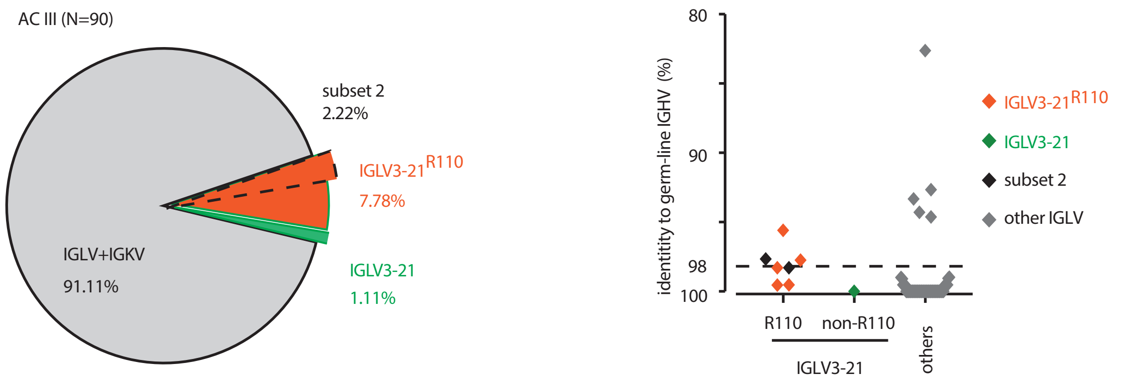

On the left, Pie chart of the immunophenotyping results depicting the proportion of IGLV3-21 (green part) and IGLV3-21^{R110} (orange part) positive cases including CLL subset#2 (dashed part) in AC III (N=90). Right, scatter plot shows IGHV mutational status of AC II CLL cases groped by different IGLV segments as follows: IGLV3-21^{R110} –positive cases (orange), IGLV3-21–positive cases (green) and others (gray). The dashed line indicates the conventional distinction of 98% identity between UM and M-CLL.

Fig. S5

Single cell immunophenotyping of IGLV3-21^{R110} CLL cases by mass-cytometry

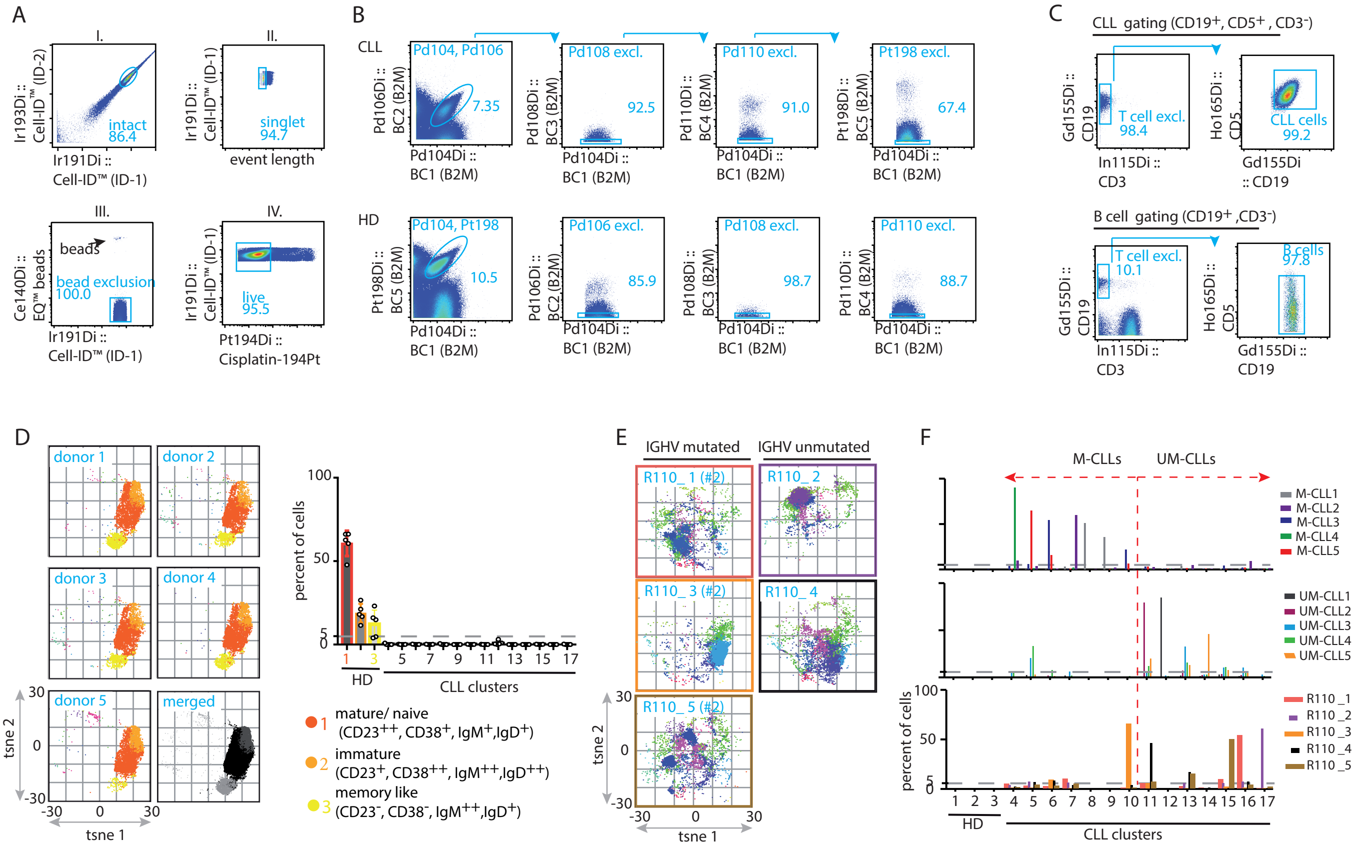

Panel A shows the CyTOF data processing and gating strategies in 4 steps (I-IV) corresponds to intact cell identification, singlet separation, bead exclusion and live cells annotation, respectively.

Panel B shows exemplary debarcoding scheme for identifying a CLL sample (top) and a HD sample (bottom) from a multiplexed CyTOF data, which concurrently measured 10 independent samples distinguished by combinatorial anti-B2M labeling.

Panel C shows gating strategy for identifying CLL cells (top) and B cells form HD (bottom). Panels A-C, all gating criteria and the frequencies inside the gate are indicated within the plot.

Panel D, left shows PhenoGraph analyses of peripheral B cells from five independent HDs and merged data set identifying three phenotypic clusters (1, 2 and 3) resembling mature/ naive (CD23^{++}, CD38^{+}, IgM^{+} and IgD^{+}), immature (CD23^{+}, CD38^{++}, IgM^{++} and IgD^{++}) and memory like (CD23^{-}, CD38^{-}, IgM^{++} and IgD^{+}) B cells. Panel D, right represents interleaved bar graph showing distribution of B cells into three different phenotypic clusters 1, 2 and 3. In all case, the surface markers CD19, CD5 and CD3 were used only for gating of the B cells (as shown in Panel C) and were excluded for PhenoGraph analyses.

Panel E shows PhenoGraph analyses of five independent IGLV3-21^{R110} CLL samples (R110) classified as IGHV mutated (left column) and IGHV unmutated (right column) cases. Notably, the stereotypic CLL subset#2 cases were found to be M-CLL cases ad indicated within the plot.

Panel F represents interleaved bar graph showing distribution of different phenotypic clusters of M-CLL (top), UM-CLL (middle) and R110 CLL (bottom) cases.

Fig. S6

Selective enrichment of IGLV3-21^{R110} expressing cells

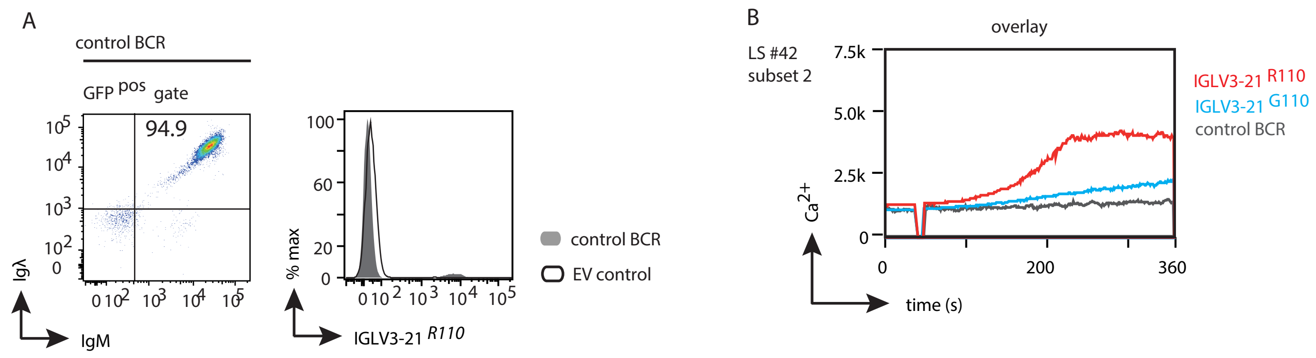

Panel A, left shows BCR expressions (IgM HC and Igλ LC) in TKO cells reconstituted with non-CLL BCR used as control (as in Fig. 6A). Right, overlaid histogram shows analysis of IGLV3-21^{R110} expression in the same cells compared to EV transfected control.

Panel B shows exemplary median Ca2+ release kinetics of CLL subset#2 derived BCRs overlaid for reverted IGLV3-21^{R110} (red) and IGLV3-21^{G110} (blue) compared to non-CLL control (as in Fig. 6B).

Fig. S7

R110 mutated LC allele *IGLV3-21*01* boosts autonomous signaling

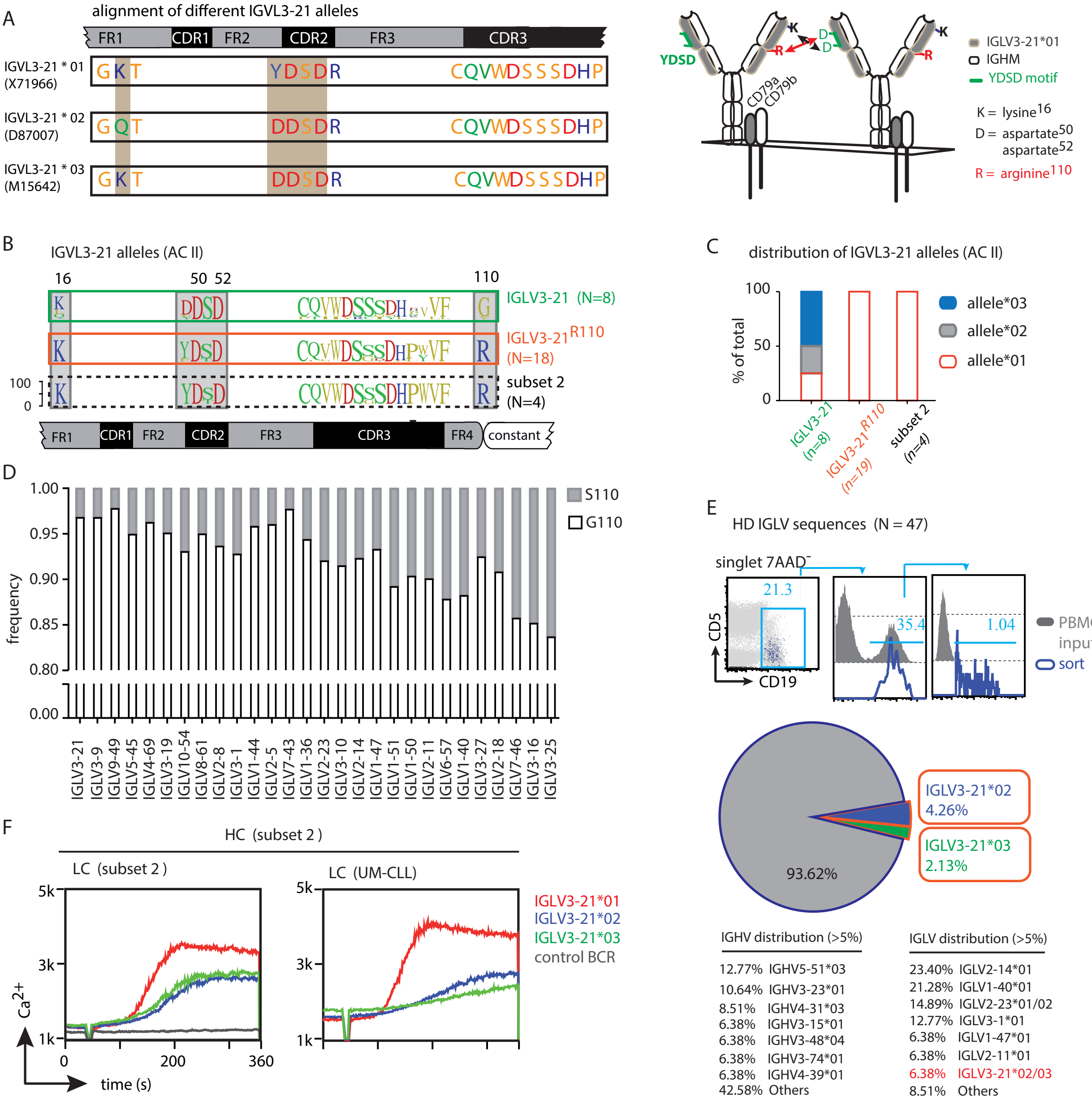

Panel A, left shows sequence alignment of three annotated IGLV3-21 alleles from the IMGT reference library, revealing the inter-allelic differences in YDSD motif and the K16 residue (brown shaded), those are involved in BCR-BCR homotypic interactions and autonomous signaling. Right, schematic representation shows BCR-BCR homotypic interaction between light chain (LC) alleles *IGLV3-21*01* carrying a mutated arginine (R) at position 110 (R110), marked in red. Other interacting allele specific unmutated amino acid (AA) residue and motif (YDSD) are indicated in black and green, respectively.

Panel B shows alignment of LC consensus sequences derived from different groups IGLV3-21, IGLV3-21^{R110} CLL cases and CLL subset#2 cases (all from AC II) compared to the reference CLL subset#2 LC.

Panel C shows stacked bar graph of three different IGLV3-21 allele frequencies in IGLV3-21 and IGLV3-21^{R110} –expressing CLL patients compared to stereotypic CLL subset#2 within AC II patients.

Panel D shows cumulative stacked frequencies of S110 (gray bars) and germ-line unmutated G110 (open bars) within the non-R110 pool (as in Fig. 7C) of different IGLV genes obtained from six HDs.

Panel E shows the gating strategy and selection of single cells from HD samples for sorting prior to IGHV and IGLV sequencing. Pie chart shows exemplary of single cell IGLV sequencing results from HD samples, revealing the frequency of IGLV3-21^{R110} expressing B cells. Usage of different IGHV and IGLV sequences obtained from this analysis were listed below the Pie chart.

Panel F shows overlaid median Ca^{2+} release kinetics of an exemplary CLL subset#2 and an exemplary UM-CLL (LS#83) derived LC allele *IGLV3-21*01* (red) compared to rendered alleles *IGLV3-21*02* (blue) and allele *IGLV3-21*03* (green), and a non-CLL BCR (gray) as control.

Table S1

Disease characteristics of 122 CLL patients with informative follow-up (AC I)

**Characteristic**	**All CLL patients**	**IGLV3-21^{R110} CLL**	**UM-CLL (non-IGLV3-21^{R110})**	**M-CLL (non-IGLV3-21^{R110})**
No. of patients (%)	122 (100)	26 (21.3)	53 (43.4)	43 (35.3)
Age –years (range)	58.1 (30.3-84.1)	55.1 (35.3-75.8)	56.7 (35.8-83.0)	62.3 (30.3-84.1)
Gender (m/f)	84/38	20/6	15/38	26/17
Rai stage – no(%)				
0	39 (32.0)	4 (15.4)	14 (26.4)	21(48.8)
1	24 (19.7)	9 (34.6)	10 (18.9)	5 (11.6)
2	11 (9.0)	3 (11.5)	5 (9.4)	3 (7.0)
3	5 (4.1)	1 (3.5)	3 (5.7)	1 (2.3)
4	10 (8.2)	3 (11.5)	5 (9.4)	2 (4.7)
unknown	33 (27.1)	6 (23.1)	17 (32.1)	11 (25.6)
FISH aberrations –no. (%)				
None	18 (14.8)	4 (9.3)	10 (18.9)	4 (9.3)
Del13q14	62 (50.1)	19 (73.1)	17 (32.1)	26 (60.1)
+12	7 (5.7)	0 (0)	6 (11.3)	1 (2.3)
Del17p	4 (3.3)	0 (0)	3 (5.7)	1 (2.3)
Del11q22	11 (9.0)	1 (3.5)	9 (17.0)	1 (2.3)
unknown	31 (25.4)	0(0)	18 (34.0)	13 (30.2)
Median Follow-up –months (range)	97.1 (0.0-326.0)	120.8 (27.8-211.5)	87.5 (0.0-304.8)	95.5 (3.7-326.0)

Table S2
Molecular characterization of 26 IGLV3-21^{R110}-expressing CLLs of AC I by ARTISAN PCR/Sanger sequencing, fluorescence in-situ hybridization on interphase nuclei, and targeted panel sequencing.

					FISH				Single Nucleotide Variants				
Sample	IGHV	% GL Id	M/UM	Subtype 2	del13q14	del17p	del11q22	+12	TP53	ATM	SF3B1	NOTCH1	Other genes
2	3-48*01	96.53	M		1	0	0	0	wt	P1054R	wt	wt	EP300 M978R<br>ZFAT R484W (R534W)
5	4-38-2*01	97.92	M		0	0	0	0	wt	wt	wt	fs2514	KMT2D P2557L<br>NOTCH2 L2408H
17	4-59*01	98.95	UM		0	0	0	0		P1054R	wt	wt	wt
19	3-48*03	99.31	UM		0	0	0	0	G66C (G105C)	F2732L	G742D	wt	EP300 P784L
21	3-23*04	98.61	UM		0	0	0	0	wt	wt	wt	wt	KMT2D I1450V<br>KMT2D P2382S
23	3-23*01 /<br>3-23D*01	96.53	M		1	0	0	0	wt	E2444K	K700E	wt	EP300 M978R<br>ZFAT E1036K (E1086K / E1098K)
27	3-15*01	99.32	UM		1	0	0	0	wt	wt	K700E	wt	CDKN2A A148T
42	3-21*01	97.57	M	Y	1	0	0	0	wt	wt	wt	fs2514	MYC S161L<br>PIM1 E215Q (E124Q)
56	3-53*01	98.25	UM		1 (hom)	0	0	0	wt	wt	wt	fs2514	wt
58	3-15*01	97.96	M		1	0	0	0	wt	V613L	G742D	wt	wt
66	3-66*02	97.89	M		1 (64% hom;27% het)	0	0	0	Y31D (Y4D / Y124D / Y163D)<br>G113S (G86S / G206S / G245S)	wt	K700E	wt	EP300 S697R
70	3-21*02	94.79	M		0	0	0	0	V84M (V57M / V177M / V216M)	wt	K700E	wt	EP300 M978R<br>MYC N26S
83	3-48*04	99.31	UM		1	0	0	0	wt	wt	N626I	fs2514	EP300 M978R<br>MYC N26S<br>PTEN F278L (F81L / F451L)<br>ZFAT R484W (R534W)<br>CXCR4 fs324
95	3-21*01	96.18	M	Y	1 (hom)	0	1	0	wt	wt	wt	wt	EP300 M978R<br>KMT2D P3665A
99	3-21*01	94.44	M	Y	1 (30% hom;53% het)	0	0	0	wt	wt	wt	wt	CREBBP V781M (V819M)
103	3-48*03	98.61	UM		1 (hom)	0	0	0	wt	wt	wt	wt	PTEN F278L (F81L / F451L)
121	3-48*03	98.61	UM		1	0	0	0	C110Y (C83Y / C203Y / C242Y)	R2832H	wt	wt	MYC N26S<br>PRDM1 S220N (S354N)
123	3-21*01	96.18	M	Y	1	0	0	0	wt	wt	wt	wt	EP300 M978R<br>MYD88 V172F (V217F)<br>SGK1 Q66E
134	3-30*03 /<br>3-30*18 /<br>3-30-5*01	97.92	M		1	0	0	0	wt	wt	wt	wt	EP300 M978R
142	3-13*01	99.65	UM		1	0	0	0		Q2714X	Y623C	wt	wt
143	3-48*02	98.26	UM		1	0	0	0	E204X (E177X / E297X / E336X)	wt	wt	wt	BORCS8-MEF2B; MEF2B P128H
145	3-21*01	98.26	UM	Y	0	0	0	0	wt	wt	K700E	wt	CREBBP S855L (S893L)<br>EP300 V520G<br>ETV6 R127Q
146	3-21*01	96.18	M	Y	1	0	0	0	wt	wt	K700E	wt	EP300 V520G
147	3-48*03	96.53	M		1	0	0	0	wt	wt	wt	wt	CDKN2A A148T
148	3-48*03	99.31	UM		1	0	0	0	wt	wt	K700E	wt	PTEN F278L (F81L / F451L)
154	3-15*01	98.64	UM		0	0	0	0	wt	D1853V	wt	wt	wt
Total:			13/13	6/26<br>(23%)	19/26<br>(73%)	0/26	1/26<br>(4%)	0/26	5/26<br>(19%)	8/26<br>(31%)	11/26<br>(42%)	4/26<br>(15%)	

Nonsynonymous mutations are specified according to their predicted amino acid alterations. Only mutations with probable or definitive pathogenic potential (class 4 / 5 mutations) are listed. M/UM: categorization of IGHV mutational status with a cut-off of 98% homology to the germ-line sequence. Y: Assignment to CLL stereotype 2 according to IGHV3-21 expression, a CDR3 length of 9 amino acids with a D or E residue at position 3, and usage of IGHJ6 [2]. FISH: Fluorescence in situ hybridization on interphase nuclei. 1: present, 0: not detectable. SNV: Single nucleotide variants. wt: wild-type. Fs: frame shift mutation.

Table S3

Analysis of potential prognostic factors for time from diagnosis to first treatment (TTFT) by log-rank and Cox regression analysis (AC I; N=91)

			**Log rank**			**Univariable Cox regression**			**Multivariable analysis**			**Multivariable analysis** (without stage)		
**Variable**	**Analysis**	**n**	**HR**	**95% CI**	**p**	**HR**	**95% CI**	**p**	**HR**	**95% CI**	**p**	**HR**	**95% CI**	**p**
**BCR**	UM-CLL vs. M-CLL	35 vs. 30	3.68	2.06-6.55	<0.0001	4.47	2.30-8.69	<0.001	2.55	1.21-5.36	0.014	3.06	1.44-6.52	0.003
	IGLV3-21^{R110} vs. M-CLL	26 vs. 30	3.12	1.61-6.07	0.0002	3.50	1.75-7.03	<0.001	3.06	1.50-6.27	0.002	3.33	1.64-6.74	<0.001
**Del13q14 (sole)**	Present vs. absent	49 vs. 42	0.41	0.25-0.67	<0.0001	0.42	0.25-0.68	<0.001	0.43	0.24-0.76	0.004	0.53	0.30-0.92	0.024
**Del17p**	Present vs. absent	4 vs. 87	3.75	0.58-24.46	0.0073	4.50	1.58-12.9	0.005	6.44	2.06-20.1	0.001	5.67	1.81-17.78	0.003
**Del11q22 (no del17p)**	Present vs. absent	10 vs. 81	1.52	0.69-3.33	0.2	1.50	0.77-2.94	0.236	1.54	0.73-3.23	0.254	1.21	0.58-2.50	0.609
**Rai stage**	2-4 vs. 0-1	19 vs. 51	3.67	1.62-8.33	<0.0001	3.99	2.17-7.32	<0.001	4.07	2.16-7.66	<0.001		(excluded)	
	Unknown vs. 0-1	21 vs. 51				2.01	1.15-3.51	0.014	1.85	1.00-3.43	0.051			
**Age**	Continuous	Median 57.2 (30.3-83.0)				0.99	0.97-1.01	0.209	1.00	0.98-1.02	0.995	0.99	0.97-1.02	0.631

Table S4

Analysis of potential prognostic factors for overall survival (OS) by log-rank and Cox regression analysis (AC I; N=90)

			Log rank			Univariable Cox regression			Multivariable analysis			Multivariable analysis (without stage)		
**Variable**	**Analysis**	**n**	**HR**	**95% CI**	**p**	**HR**	**95% CI**	**p**	**HR**	**95% CI**	**p**	**HR**	**95% CI**	**p**
**BCR**	UM-CLL vs. M-CLL<br>IGLV3-21^{R110} vs. M-CLL	35 vs. 30<br>25 vs. 30	7.36<br>5.97	3.36-16.12<br>2.29-15.55	0.0001<br>0.0009	7.86<br>6.05	2.35-26.36<br>1.71-21.42	<0.001<br>0.005	8.00<br>4.42	2.01-31.81<br>1.18-16.51	0.003<br>0.027	7.29<br>5.59	1.81-29-27<br>1.53-20.38	0.005<br>0.009
**Del13q14 (sole)**	Present vs. absent	48 vs. 42	0.51	0.27-0.95	0.035	0.72	0.38-1.37	0.311	0.93	0.42-2.07	0.856	0.88	0.39-2.01	0.768
**Del17p**	Present vs. absent	4 vs. 86	4.38	0.43-44.55	0.053	4.70	1.37-16.13	0.138	2.44	0.64-9.38	0.193	2.63	0.67-9.77	0.169
**Del11q22 (no del17p)**	Present vs. absent	10 vs. 80	2.68	0.89-8.14	0.0074	2.88	1.29-6.42	0.010	2.10	0.78-5.65	0.141	1.72	0.66-4.52	0.268
**Rai stage**	2-4 vs. 0-1<br>Unknown vs. 0-1	19 vs. 51<br>20 vs. 51	1.67	0.71-3.89	0.084	1.70<br>0.65	0.79-3.65<br>0.28-1.52	0.176<br>0.319	3.03<br>0.79	1.29-7.14<br>0.29-2.14	0.011<br>0.644		(excluded)	
**Age**	Continuous	Median 57.2 (30.3-83.0)				1.05	1.02-1.08	<0.001	1.06	1.03-1.10	<0.001	1.06	1.03-1.10	<0.001

Table S5

Analysis of potential prognostic factors for time from diagnosis to first treatment (TTFT) by Cox regression analysis (AC I; N=91)

			**Univariable Cox regression**			**Multivariable analysis**			**Multivariable analysis** (without stage)		
**Variable**	**Analysis**	**n**	**HR**	**95% CI**	**p**	**HR**	**95% CI**	**p**	**HR**	**95% CI**	**p**
**BCR**	UM-CLL vs. M-CLL	35 vs. 30	7.86	2.35-26.35	<0.001	2.51	1.19-5.31	0.016	3.08	1.45-6.58	0.004
	UM-IGLV3-21^{R110} vs. M-CLL	13 vs. 30	5.96	1.46-24.25	0.013	2.84	1.22-6.58	0.015	3.52	1.55-7.99	0.003
	M-IGLV3-21^{R110} vs. M-CLL	13 vs. 30	6.12	1.60-23.43	0.009	3.32	1.44-7.65	0.005	3.15	1.38-7.20	0.007
**Del13q14 (sole)**	Present vs. absent	49 vs. 42	0.42	0.25-0.68	<0.001	0.42	0.23-0.75	0.004	0.53	0.30-0.93	0.028
**Del17p**	Present vs. absent	4 vs. 87	4.50	1.58-12.9	0.005	6.54	2.09-20.52	0.001	5.63	1.79-17.66	0.003
**Del11q22 (no del17p)**	Present vs. absent	10 vs. 81	1.50	0.77-2.94	0.236	1.53	0.73-3.21	0.265	1.22	0.59-2.53	0.597
**Rai stage**	2-4 vs. 0-1	19 vs. 51	3.99	2.17-7.32	<0.001	4.12	2.18-7.79	<0.001		(excluded)	
	Unknown vs. 0-1	21 vs. 51	2.01	1.15-3.51	0.014						
**Age**	Continuous	Median 57.2 (30.3-83.0)	0.99	0.97-1.01	0.209	1.00	0.98-1.02	0.995	1.00	0.97-1.02	0.672

Table S6

Analysis of potential prognostic factors for overall survival (TTFT) by Cox regression analysis (AC I; N=90)

			Univariable Cox regression			Multivariable analysis			Multivariable analysis (without stage)		
**Variable**	**Analysis**	**n**	**HR**	**95% CI**	**p**	**HR**	**95% CI**	**p**	**HR**	**95% CI**	**p**
**BCR**	UM-CLL vs. M-CLL	35 vs. 30	7.86	2.35-26.35	<0.001	8.24	2.06-33.01	0.003	7.51	1.86-30.33	0.005
	UM-IGLV3-21^{R110} vs. M-CLL	12 vs. 30	5.96	1.46-24.25	0.013	5.19	1.15-23.52	0.032	6.78	1.57-29.37	0.011
	M-IGLV3-21^{R110} vs. M-CLL	13 vs. 30	6.12	1.60-23.43	0.009	4.06	1.02-16.21	0.047	5.01	1.28-19.68	0.021
**Del13q14 (sole)**	Present vs. absent	48 vs. 42	0.72	0.38-1.37	0.311	0.98	0.42-2.24	0.952	0.92	0.40-2.12	0.851
**Del17p**	Present vs. absent	4 vs. 86	4.70	1.37-16.13	0.138	2.39	0.62-9.22	0.204	2.54	0.66-9.70	0.174
**Del11q22 (no del17p)**	Present vs. absent	10 vs. 80	2.88	1.29-6.42	0.010	2.10	0.78-5.66	0.144	1.75	0.66-4.63	0.260
**Rai stage**	2-4 vs. 0-1	19 vs. 51	1.70	0.79-3.65	0.176	2.96	1.25-7.02	0.014		(excluded)	
	Unknown vs. 0-1	20 vs. 51	0.65	0.28-1.52	0.319	0.77	0.28-2.09	0.604			
**Age**	Continuous	Median 57.2 (30.3-83.0)	1.05	1.02-1.08	<0.001	1.06	1.03-1.10	<0.001	1.06	1.03-1.10	<0.001

Table S7

Disease characteristics of 134 CLL (IGLV) patients with informative follow-up (AC II)

Characteristic	All CLL (IGLV) patients	IGLV3-21^{R110} CLL	IGLV3-21 CLL	UM-CLL (non-IGLV3-21)	M-CLL (non-IGLV3-21)
**No. of patients (%)**	134 (100)	23 (17.16)	10 (7.46)	35 (26.11)	66 (49.25)
**Age –years (range)**	65 (33-84)	66 (52-84)	64 (33-84)	60 (39-84)	65 (45-80)
**Gender (m/f)**	85/49	20/3	7/3	25/10	66/0
**Rai stage – no**					
**0**	36	6	2	3	25
**1**	63	11	6	18	28
**2**	20	3	2	8	7
**3**	3			1	2
**4**	5	3		1	1
**unknown**	7			4	3
**FISH aberrations –no. (%)**					
**None**	31 (14.8)	8 (34.8)	5 (50.0)	5 (14.3)	13 (19.7)
**Del13q14**	57 (42.5)	10 (43.5)		6 (17.1)	41 (62.1)
**+12**	10 (7.5)		1 (10.0)	4 (11.4)	5 (7.6)
**Del17p**	20 (14.9)		1 (10.0)	12 (34.3)	7 (10.6)
**Del11q22**	15 (11.2)	5 (21.7)	3 (30.0)	7 (20.0)	
**unknown**	1 (0.7)			1 (2.9)	
**Median Follow-up –months (range)**	73 (0-269)	43 (2-181)	55 (16-225)	71 (0-217)	88.5 (4-269)

Table S8

Detail characterization of 23 IGLV3-21^{R110}-expressing CLL cases from AC II: Age, Rai stage, IGHV mutation, FISH aberrations and TP53 /NOTCH1 mutations.

ID	Age (>65)	RAI stage	% IGHV mutation	Category (M/UM)	IGHV	HCDR3	Subset #2	FISH		TP53 mutation	NOTCH1 mutation
								Del13q14	Del11q22		
CT336	0	0	97.44	M	3-21	DRNAMDV	Y	1	0	0	0
CT346	1	0	97.45	M	3-23	AKQNGDYGGPIDF		0	1	0	0
RM1	1	0	96.94	M	3-48	EGDNYKNAAWDY		1	0	0	0
RMPTV151	1	0	96.26	M	3-48	PIQQTMVREY		1	0	0	0
TS107	1	0	96.6	M	3-64	HESPHSSSAGAFDY		0	0	0	1
TS64	1	0	95.58	M	3-23	GGETERDVDTMAGAFDI		0	1	1	0
CT119	1	I	97.25	M	3-13	RGVTTYAFDI		0	0	0	0
CT307	1	I	94.56	M	3-48	EGYDSSGYRLY		0	0	0	0
CT81	1	I	96.6	M	3-23	AGSSSSGAFDY		1	0	0	0
RM599	1	I	98	M	3-15	GESDLSDY		0	1	0	0
RMPTV327	0	I	97.6	M	3-21	DRSGMDV	Y	1	0	0	0
RMPTV40	1	I	97.09	M	3-21	FVEQLATDV		1	0	0	0
RM395	1	II	97.98	M	3-15	DICSSTSCFD		0	1	0	1
RMPTV184	1	IV	96.22	M	4-34	LDVVVVATTPCNWFDP		0	0	0	0
UD4	0	IV	97.07	M	3-21	DQNGMDV	Y	0	0	0	0
CT324	0	I	98.64	UM	3-48	DPPTPSPEYS		1	0	0	0
RM391	0	I	99.32	UM	3-30	DDPSSIDP		1	0	0	0
RMPTV209	1	I	98.26	UM	3-21	DANHMDV	Y	0	0	0	0
RMPTV296	0	I	98.26	UM	3-30	AKGEEVSLALGRSYDSSGYYDY		1	0	0	0
RM274	1	II	98.64	UM	3-11	DHEG		0	1	0	0
RMPTV343	1	II	99.31	UM	4-59	GPVDRADY		0	0	0	1
RMPTV137	0	IV	99	UM	3-15	CTTDSFLW		1	0	0	1
RM470	0	I	98.98	UM	3-7	GYGSGSYYNSPFDY		0	0		
**Total (%)**	**15/23 (65.2)**							**10/23 (43.5)**	**5/23 (21.7)**	**1/23 (4.3)**	**4/23 (17.4)**

1: positive / present, 0: 0 / absent. Y: Assignment to CLL subset #2 stereotype

Table S9

Disease characteristics of 90 high-risk CLL patients with informative follow-up (AC III)

Characteristic	All patients	IGLV3-21^{R110} CLL	IGLV3-21 CLL	UM-CLL (non-IGLV3-21)	M-CLL (non-IGLV3-21)
**No. of patients (%)**	90 (100)	7 (7.8)	1 (1.1)	76 (84.4)	5 (5.6)
**Age –years (range)**	66 (36-79)	62(54-70)	72	64 (36-79)	68 (51-73)
**Gender (m/f)**	66/24	6/1	1/0	57/19	3/2
**Binet stage – no (%)**					
**A**	9 (10)	2 (28.6)	0	7 (9.2)	0
**B**	29 (32.2)	1 (14.3)	1 (100)	24 (31.6)	2 (40)
**C**	52 (57.8)	4 (57.1)	0	44 (57.9)	3 (60)
**FISH aberrations –no. (%)**					
**None**	2 (2.2)	0	0	2 (2.6)	0
**Del13q14**	52 (57.8)	6 (85.7)	0	21 (27.6)	5 (100)
**+12p11**	14 (15.6)	0	1 (100)	5 (6.6)	1 (20)
**Del17p13**	76 (84.4)	2 (28.6)	1 (100)	37 (48.7)	5 (100)
**Del14q32**	13 (14.4)	2 (28.6)	1 (100)	5 (6.6)	0
**Del11q22**	15 (16.7)	1 (14.3)	0	8 (10.5)	0
**Allo-PBSCT**	24	1	0	11	0
**Median Follow-up –months (range)**	*				

***3 year follow up period for all 90 patients are available**

Table S10

Detail characterization of 7 IGLV3-21^{R110}-expressing CLL cases from AC III: IGHV mutation, subset assignment, FISH aberrations and mutations.

ID	% IGHV identity	(M/ UM)	IGHV	IGHD	IGHJ	Subset	Allo-PBSCT	FISH				Mutations		
								**Del13q14**	**+12**	**Del17p**	**Del11q22**	**TP53**	**NOTCH1**	**SF3B1**
**08PB4641**	97.75	M	V3-21	-	JH6b	2	0	1	0	0	0	0	0	0
**09PB2740**	97.65	M	V3-21	D4-23	JH4b	-	0	1	0	1	0	1	0	1
**10PB7034**	95.59	M	V4-59	D 3-22	JH3b	-	0	1	0	0	0	1	0	1
**09PB8021**	99.52	UM	V3-66	D3-22	JH3b	-	1	0	0	1	0	1	0	0
**10PB6155**	99.54	UM	V3-15	D 6-19	JH4b	-	0	1	0	0	0	1	0	0
**10PB6260**	98.30	UM	V3-21	D 1-1	JH6b	2	0	1	0	0	1	1	0	0
**11PB2028**	98.30	UM	V3-48	D 3-22	JH4b	169	0	1	0	0	0	0	0	1
Total							**1/7**	**6/7**	**0/7**	**2/7**	**1/7**	**5/7**	**0/7**	**3/7**

1: positive / present, 0: 0 / absent.

Table S11

List of CLL samples and related characteristics of Analysis cohort IV

Group	Patient ID	Rai Stage	M/ UM	% IGHV Identity	IGHV	IGLV	Subset	FISH				Mutations	
								Del13q14	+12	Del11q22	Del17p	NOTCH1	TP53
IGLV3-21	23328	0	M	90.51	IGHV3-23*01	IGLV3-21*01		1	0	0	0	0	0
	30034	0	M	92.01	IGHV3-30*03	IGLV3-21*02		1	0	0	0	0	0
	12452	I	M	95.88	IGHV2-5*04	IGLV3-21*01		1	0	0	0	0	0
	6540	IV	M	96.2	IGHV3-48*02	IGLV3-21*01		0	0	0	0	0	NA
	7209	0	M	91.2	IGHV3-53*01	IGLV3-21*01		0	0	0	0	0	NA
	6938	0	UM	100	IGHV3-30*01	IGLV3-21*03		1	0	0	0	0	0
	7395	I	UM	100	IGHV3-30*04	IGLV3-21*02		0	0	0	0	0	0
	11429	0	UM	100	IGHV3-30*01	IGLV3-21*01		0	0	0	0	0	1
IGLV3-21^{R110}	4562	I	M	95.1	IGHV3-48*03	IGLV3-21*02		NA	NA	NA	NA	0	0
	12531	0	M	96.14	IGHV4-4*07	IGLV3-21*01		NA	NA	NA	NA	0	NA
	35712	NA	M	96.53	IGHV3-21*01	IGLV3-21*01	2	NA	NA	NA	NA	NA	NA
	10303	II	M	96.91	IGHV3-23*01	IGLV3-21*01		1	0	0	0	0	NA
	12836	I	M	97.2	IGHV3-20*01	IGLV3-21*01		1	0	0	1	0	1
	16021	NA	M	97.22	IGHV3-21*01	IGLV3-21*01	2	NA	NA	0	0	NA	NA
	8154	0	M	97.4	IGHV3-7*01	IGLV3-21*01		0	0	0	0	0	NA
	18620	I	M	97.6	IGHV3-48*01	IGLV3-21*01		0	0	0	0	0	NA
	6193	NA	M	97.9	IGHV3-48*02	IGLV3-21*01		1	0	0	0	0	NA
	31309	I	M	97.92	IGHV3-21*01	IGLV3-21*01	2	0	0	1	0	0	0
	11843	0	M	97.99	IGHV3-21*01	IGLV3-21*01	2	0	0	1	0	0	0
	4957	NA	UM	98.3	IGHV3-48*01	IGLV3-21*02		NA	NA	NA	NA	0	NA
	21735	II	UM	98.96	IGHV3-48*02	IGLV3-21*01	169	0	0	0	1	NA	NA
	33949	II	UM	100	IGHV2-26*01	IGLV3-21*01		1	0	0	0	0	1

1: positive / present, 0: 0 / absent, NA: not available,

Table S12

List of CLL samples and related characteristics of Analysis cohort V

Group	Patient ID	Rai Stage	M/ UM	% IGHV Identity	IGHV	IGLV	Subset	FISH				Mutations	
								Del13q14	+12	Del11q22	Del17p	NOTCH1	TP53
**IGLV3-21**	CLL1631	0	M	90.51	IGHV3-23*01	IGLV3-21*01		1	0	0	0	0	0
**IGLV3-21^{R110}**	CLL105	IV	UM	98.3	IGHV3-23*01	IGLV3-21*01	NA	NA	NA	NA	NA	NA	NA
	CLL229	III	UM	99.3	IGHV3-48*01	IGLV3-21*01	169	1	0	1	0	NA	NA
	CLL335	I	UM	98.3	IGHV3-21*01	NA	2	1	0	1	0	NA	NA
	CLL715	I	UM	98.6	IGHV3-21*01	NA	2	1	0	0	0	NA	NA
	CLL785	NA	UM	98.6	IGHV3-21*01	IGLV3-21*01	2	1	0	0	0	NA	NA
	CLL1735	0	UM	99.0	IGHV3-21*01	NA	2	1	0	0	0	NA	NA
	CLL237	III	M	97.5	IGHV3-21*01	IGLV3-21*01	2	1	0	0	0	NA	NA
	CLL306	II	M	96.2	IGHV3-21*01	NA	2	1	0	0	0	NA	NA
	CLL668	I	M	97.6	IGHV3-21*01	IGLV3-21*01	2	1	0	0	0	NA	NA
	CLL707	NA	M	95.8	IGHV3-21*01	NA	2	NA	NA	NA	NA	NA	NA
	CLL853	II	M	97.9	IGHV3-21*01	IGLV3-21*01	2	0	0	0	0	NA	NA
	CLL1518	NA	M	96.2	IGHV3-21*01	NA	2	0	0	0	0	NA	NA
	CLL1747	0	M	97.2	IGHV3-21*01	NA	2	1	0	1	0	NA	NA
	CLL1929	NA	M	96.5	IGHV3-21*02	NA	2	1	0	0	0	NA	NA

1: positive / present, 0: 0 / absent, NA: not available,

Table S13

List of CLL samples used in mass-cytometry (Cytof) analyses and related characteristics

Gr	Patient ID	FISH			Gene mutations			IGHV (%)	IGHV	IGHD	IGHJ	CLL #2	IGL/ IGK	IGLV	IGLJ
		del13q14	del11q22	del17p13	TP53	NOTCH1	SF3B1								
M-CLL	**15PB19296**	1 (het)	0	0	0	0	0	90.97	IGHV4-4*02	IGHD2-2*01	IGHJ4*02		IGK	n.d.	n.d.
	**15PB20048**	1 (het, hom)	0	0	0	0	0	92.16	IGHV4-34*01	IGHD5-18*01	IGHJ4*02		IGL	n.d.	n.d.
	**15PB20475**	1 (het)	0	0	0	0	1	93.72	IGHV3-7*02	IGHD4-23*01	IGHJ4*02		IGL	IGLV3-21*01	IGLJ2*01
	**16PB2203**	1 (het, hom)	0	0	n.d.	n.d.	n.d.	90.87	IGHV4-34*02	IGHD2-15*01	IGHJ3*02		IGK	n.d.	n.d.
	**17PB2915**	1 (het)	1 (het)	0	n.d.	n.d.	n.d.	90.16	IGHV2-5*02	IGHD3-10*01	IGHJ4*02		IGL	IGLV3-21*02	IGLJ3*02
UM-CLL	**16PB11311**	1 (het)	0	0	0	1	0	100	IGHV1-69*01	IGHD3-10*01	IGHJ4*02		IGK	n.d.	n.d.
	**16PB11526**	1 (het)	1(het)	0	0	0	0	100	IGHV4-30-4*01	IGHD3-3*01	IGHJ4*02		IGL	n.d.	n.d.
	**16PB12343**	0	0	0	0	1	0	100	IGHV2-5*02	IGHD3-3*01	IGHJ5*02		IGK	n.d.	n.d.
	**16PB12783**	1 (het)	0	1 (het)	1	0	0	100	IGHV1-46*01	IGHD3-3*01	IGHJ4*02		IGK	n.d.	n.d.
	**17PB9458**	no	1 (het)	0	n.d.	n.d.	n.d.	100	IGHV3-23*01	IGHD3-22*01	IGHJ3*02		IGL	IGLV3-21*01	IGLJ2*01
R110	**15PB18924**	0	0	0	n.d.	n.d.	n.d.	97.06	IGHV3-21*01	IGHD2-15*01	IGHJ6*02	Y	IGL	IGLV3-21*01	IGLJ3*02
	**16PB9582**	1 (het, hom)	0	0	1	0	1	96.62	IGHV3-21*01	IGHD1-26*01	IGHJ6*02	Y	IGL	IGLV3-21*02	IGLJ3*02
	**16PB11833**	1 (het)	1 (het)	0	0	0	1	96.09	IGHV3-21*01	IGHD5-24*01	IGHJ6*02	Y	IGL	IGLV3-21*01	IGLJ3*02
	**15PB19100**	0	0	0	n.d.	n.d.	n.d.	98.25	IGHV1-3*01	IGHD6-13*01	IGHJ4*02		IGL	IGLV3-21*02	IGLJ1*01
	**16PB4117**	0	0	0	n.d.	n.d.	n.d.	98.26	IGHV3-23*01	IGHD6-6*01	IGHJ1*01		IGL	IGLV3-21*01	IGLJ3*02

1: positive / present, 0: 0 / absent, n.d. : not determined, Y: Assignment to CLL subset #2 stereotype
